# Cancer Cell Growth Is Differentially Affected by Constitutive Activation of NRF2 by KEAP1 Deletion and Pharmacological Activation of NRF2 by the Synthetic Triterpenoid, RTA 405

**DOI:** 10.1371/journal.pone.0135257

**Published:** 2015-08-24

**Authors:** Brandon L. Probst, Lyndsey McCauley, Isaac Trevino, W. Christian Wigley, Deborah A. Ferguson

**Affiliations:** Department of Research, Reata Pharmaceuticals Inc., Irving, Texas, United States of America; Columbia University, UNITED STATES

## Abstract

Synthetic triterpenoids are antioxidant inflammation modulators (AIMs) that exhibit broad anticancer activity. AIMs bind to KEAP1 and inhibit its ability to promote NRF2 degradation. As a result, NRF2 increases transcription of genes that restore redox balance and reduce inflammation. AIMs inhibit tumor growth and metastasis by increasing NRF2 activity in the tumor microenvironment and by modulating the activity of oncogenic signaling pathways, including NF-κB, in tumor cells. Accumulating evidence suggests that KEAP1 loss or mutation—which results in high levels of sustained NRF2 activity—may promote cancer growth and increase chemoresistance. Loss of KEAP1 also increases the levels of other oncogenic proteins, including IKKβ and BCL2. The apparent survival advantage provided to some tumor cells by loss of functional KEAP1 raises the question of whether pharmacological inhibition of KEAP1 could promote tumor growth. To address this issue, we characterized the basal levels of KEAP1 and NRF2 in a panel of human tumor cell lines and profiled the activity of an AIM, RTA 405. We found that in tumor cell lines with low or mutant KEAP1, and in *Keap1* ^*-/-*^ murine embryonic fibroblasts, multiple KEAP1 targets including NRF2, IKKβ, and BCL2 were elevated. *Keap1* ^-/-^ murine embryonic fibroblasts also had higher rates of proliferation and colony formation than their wild-type counterparts. In cells with functional KEAP1, RTA 405 increased NRF2 levels, but not IKKβ or BCL2 levels, and did not increase cell proliferation or survival. Moreover, RTA 405 inhibited growth at similar concentrations in cells with different basal NRF2 activity levels and in cells with wild-type or mutant *KRAS*. Finally, pre-treatment with RTA 405 did not protect tumor cells from doxorubicin- or cisplatin-mediated growth inhibition. Collectively, these data demonstrate that pharmacological activation of NRF2 by AIMs is distinct from genetic activation and does not provide a growth or survival advantage to tumor cells.

## Introduction

Synthetic oleanane triterpenoids, such as bardoxolone methyl and RTA 408, are antioxidant inflammation modulators (AIMs) that exhibit broad antitumor activity in models of cancer prevention and treatment [1;2], and are well-tolerated in patients with advanced malignancies [3]. The primary target of the AIMs is Kelch-like ECH-associated protein 1 (KEAP1), a substrate adaptor protein for the CUL3-RBX1 E3 ubiquitin ligase complex. Under normal conditions, KEAP1 facilitates the ubiquitination of its substrate proteins, which leads to their degradation by the proteasome [4]. One of these substrates is the transcription factor nuclear factor (erythroid-derived 2)-like 2 (NFE2L2), also known as NRF2. AIMs potently increase NRF2 activity by binding to a sensor cysteine residue (C151) in KEAP1, causing a conformational change that renders KEAP1 unable to promote NRF2 degradation [5–8]. NRF2 accumulates and enters the nucleus where it increases the expression of antioxidant response element (ARE)-containing target genes. The products of these genes include antioxidant and cytoprotective proteins, which restore cellular redox balance and reduce inflammation. Accordingly, AIMs have demonstrated broad anti-inflammatory and cytoprotective activity in many animal models, as well as in the clinic [9;10].

The increase in antioxidant and anti-inflammatory activity that occurs upon treatment with AIMs reduces tumor incidence and burden in chemically- and genetically-induced models of carcinogenesis [11–14]. Likewise, AIMs reduce oxidative stress and inflammation in the microenvironment of established tumors and inhibit tumor growth, metastasis, angiogenesis, and tumor-mediated immune suppression [15–20]. AIMs also directly inhibit proliferation and induce apoptosis in tumor cells [1;2;21;22]. Although the mechanism underlying the direct anticancer effect of AIMs on tumor cells is not completely understood, it is known that AIMs modulate the activity of several cancer-related proteins, including cyclin D1 [12;23], CDKN1A (p21) [23], JAK1 and STAT3 [24;25], HER2 [26], and nuclear factor kappa B (NF-κB) [27–29].

Although NRF2 plays an anticancer role in the tumor microenvironment, it has been proposed to have the opposite effect in malignant cells [30;31]. Recent genetic analyses of human tumors have demonstrated that mutations in *KEAP1* or *NRF2*—which result in high levels of sustained NRF2 activity—are associated with increased proliferation, chemoresistance, and poor survival [30;31]. Other mechanisms of NRF2 activation have also been identified, including reduced *KEAP1* expression due to promoter hypermethylation or miRNA expression [32–34] and increased expression of *NRF2* due to activated oncogenes, such as KRAS [35]. It has been suggested that elevated NRF2 activity provides a survival advantage to tumor cells by increasing antioxidant levels to manage excess reactive oxygen species (ROS) and reactive nitrogen species (RNS), which are common features of cancer [35]. These observations raise the question of whether pharmacological agents that activate NRF2 via KEAP1 inhibition could promote cancer growth or increase therapeutic resistance [31;36;37]. This question is especially important given the potential of NRF2 activators to prevent and treat a variety of chronic inflammatory and autoimmune diseases [38–40].

Consistent with the overall anticancer activity of the AIMs, there is no evidence that these compounds increase the incidence of cancer in animal models [36;37]; rather, there is strong evidence to the contrary [1;31]. Therefore, genetic induction of NRF2 by loss of KEAP1 function appears to have a different effect than AIM-mediated activation of NRF2 via KEAP1 inhibition on tumor growth. However, both the NRF2-dependent effects on the tumor microenvironment and the NRF2-independent effects on the tumor cells likely contribute to the anticancer activity of the AIMs in vivo. To our knowledge, the effect of AIM-mediated NRF2 induction on the proliferation, survival, and chemosensitivity of isolated tumor cells has not previously been assessed.

To evaluate the effect of AIM-mediated NRF2 induction on tumor cell growth and survival, we first characterized the basal level of NRF2 activity in a panel of tumor cell lines to identify those that had a wild-type KEAP1-NRF2 axis (ie, low basal NRF2 levels), and those that had a dysfunctional KEAP1-NRF2 axis (ie., high basal NRF2 levels). With this information, we evaluated the anticancer activity of an AIM, RTA 405 (CDDO-Ethyl Amide) [8;11;41–47] in tumor cell lines where NRF2 activity could be induced (ie, those with a wild-type KEAP1-NRF2 axis) compared with tumor cell lines where NRF2 activity was already at its maximal level (ie, elevated NRF2 activity due to loss of KEAP1 function). To directly compare the effects of loss of KEAP1 function to the effects of pharmacological KEAP1 inhibition, we treated wild-type (WT) and *Keap1* ^-/-^ murine embryonic fibroblasts with RTA 405 and assessed proliferation and survival, as well as the levels of IKKβ and BCL2—two other KEAP1 substrates that have cancer promoting activities. We also evaluated the effect of RTA 405-mediated NRF2 activation on KRAS-induced proliferation and the sensitivity of tumor cell lines to other chemotherapeutics. Taken together, the results from this study demonstrate that NRF2 induction by AIMs does not increase cellular proliferation or survival and that the downstream effects of pharmacological KEAP1 inhibition by AIMs are distinct from those that result from the loss of functional KEAP1 in tumor cells.

## Materials and Methods

### Materials

RTA 405 (2-cyano-3,12-dioxooleana-1,9(11)-dien-28-oic acid ethyl amide) and RTA 402 (methyl 2-cyano-3,12-dioxoolean-1,9-dien-28-oate) were synthesized by Reata Pharmaceuticals, Inc. (Irving, TX USA). Unless noted, all other chemicals were purchased from Sigma-Aldrich (St. Louis MO, USA). Wild-type and *Keap1* ^-/-^ murine embryonic fibroblasts (MEFs) were obtained from Dr. Masayuki Yamamoto (Tohoku University, Japan) [48;49]. LSL-Kras^G12D^ MEFs were obtained from Dr. Tyler Jacks (Massachusetts Institute of Technology, Cambridge, MA) [50]. All other cell lines used in this study were from the American Type Culture Collection (ATCC, Manassas, VA USA). ATCC uses short tandem repeat (STR) analysis to screen all human cell lines for authenticity and purity before distribution. All cell lines were passaged for less than six months after resuscitation.

### Cell culture

MEFs, MCF-7, PANC-1, A549, and HeLa cells were cultured in Dulbecco’s Modified Eagle Medium (DMEM), SK-N-SH cells in Eagle’s Minimum Essential Medium (EMEM), and G-361 cells in McCoy’s 5A medium. DMEM and McCoy’s 5A medium were obtained from Life Technologies, Grand Island, NY USA. EMEM was obtained from ATCC, Manassas, VA USA. All other cell lines were cultured in RPMI 1640 medium (Life Technologies). Culture medium was supplemented with 10% FBS and 1% penicillin/streptomycin. FBS used in culture of LSL- Kras^G12D^ MEFs was heat-inactivated. Cell lines were cultured in the presence of 5% CO_2_ at 37°C.

### Cell growth and clonogenic assays

For cell counting experiments, MEFs (5 x 10^4^ cells/well) were plated in 6-well dishes, treated with RTA 405 the following day, and counted at various time intervals using a Vi-CELL XR cell analyzer (Beckman Coulter, Indianapolis, IN USA). For clonogenic assays, wild-type (1 x 10^3^ cells/well) and *Keap1* ^-/-^ (0.5 x 10^3^ cells/well) MEFs were seeded in 6-well dishes and, 6 hours later, treated with RTA 405. After seven days, colonies were fixed (1:7 acetic acid:methanol) and stained with 0.5% crystal violet in methanol. Colonies of ≥50 cells were counted.

To determine IC_50_ and GI_50_ values, cells were plated in 96-well plates and treated with RTA 405 at concentrations ranging from 50 to 1000 nM. Cell growth was assessed using the sulforhodamine B (SRB) assay [51]. In brief, 50 μL ice-cold 50% (w/v) trichloroacetic acid was added to each well and the plates were incubated at 4°C for 1 hour. The fixed cells were then washed five times with tap water and air-dried overnight. The following day, the cells were stained with 0.4% (w/v) SRB in 1% acetic acid at room temperature for 20 minutes. Stained cells were then washed five times with 1% acetic acid and air-dried. SRB dye was solubilized by adding 200 μL 10 mM Tris Base and absorbance was measured at 490 nm. For IC_50_ determination, cell viability was assessed after 48 hours of growth. For GI_50_ determination, cells in one plate were fixed at the start of the experiment (time 0) and cells in a duplicate plate were treated with RTA 405 for 72 hours. The percentage of growth of RTA 405-treated cells relative to vehicle-treated cells was calculated using the following equation: [(T_i_-T_z_)/(C-T_z_)] x 100, where (T_z_) is the absorbance value at time zero, (C) is absorbance value from vehicle-treated wells after 72 hours, and (T_i_) is the absorbance value from wells treated with the drug. For combination experiments with doxorubicin or cisplatin, cells were pre-treated with RTA 405 for 2, 6, or 24 hours and then treated with doxorubicin or cisplatin for an additional 72 hours. IC_50_ and GI_50_ values were determined from dose-response curves using GraphPad Prism (GraphPad Software, La Jolla, CA USA).

### Real-time reverse transcription PCR

Total RNA was isolated from cells with the RNeasy Mini Kit (Qiagen, Germantown, MD USA) and reverse transcribed using Superscript II (Life Technologies). PCR reactions were performed using primers validated for specificity and amplification efficiency. Reverse transcription and PCR cycle information can be found in S1 Protocols and PCR primer sequences are provided in S1 Table. *Ribosomal protein S9* (*RPS9*) and *ribosomal protein L19* (*Rpl19*) were used as reference genes for human and mouse samples, respectively. The relative abundance of each target gene was determined using the comparative Ct method (ΔΔCt) [52].

### *KEAP1* and *NRF2* sequencing

Genomic DNA was isolated from cells using the DNeasy kit (Qiagen). PCR amplification and sequencing of the coding exons of *KEAP1* and exon 2 of *NRF2* was performed using primers as previously described [53;54]. PCR products were purified using QIAquick PCR purification kit (Qiagen) and sequenced by Sequetech Corporation (Mountain View, CA USA). All mutations were confirmed by sequencing in both directions.

### Western blotting

Experimental details for preparation of whole cell lysates and nuclear extracts are in S1 Protocols. Protein concentration was determined using DC Protein Assay (Bio-Rad, Hercules, CA USA). Proteins (20 to 40 μg) were resolved by SDS-PAGE, and transferred to nitrocellulose membranes. Membranes were incubated with primary antibodies overnight at 4°C. Antibody information is provided in S2 Table. Horseradish-peroxidase conjugated secondary antibodies were from Jackson ImmunoResearch (West Grove, PA USA).

### ROS and glutathione assays

Basal ROS levels were measured using CM-H_2_DCFDA (Molecular Probes, Eugene, OR USA). Total glutathione levels were measured using the GSH-Glo Glutathione Assay (Promega, Madison, WI USA). Glutathione levels were normalized to cellular protein levels using the SRB assay. To control for variability between experiments, the basal ROS and total glutathione level for each cell line was normalized to NCI-H460 (set to a value of 1). Additional experimental details for ROS and glutathione assays can be found in S1 Protocols.

### Caspase-3/7 activity

Caspase-3/7 activity was determined as described previously [55] using DEVD-AFC (EMD Biosciences, Billerica, MA USA) as the substrate. To control for variability between experiments, the fluorescence of each sample was normalized to 786–0 (set to a value of 100).

### siRNA

A549, DU 145, and NCI-H460 cells were reverse transfected in OptiMEM with Lipofectamine RNAiMax (Life Technologies, Grand Island NY USA) and 20 nM siNRF2 or 20 nM siNTPool (GE Dharmacon, Lafayette, CO USA), L-003755-00-0005 and D-001810-10-05, respectively). Mock samples did not receive siRNA. Cells were plated in 24-well plates at a density of 4 x 10^5^ (A549 and DU 145) or 8 x 10^5^ (NCI-H460) cells per well or in 96-well plates at a density of 8 x 10^3^ (A549 and DU 145) or 1.6 x 10^4^ (NCI-H460) cells per well in RPMI 1640 media supplemented with 10% FBS. Twenty-four hours after transfection cells were treated with DMSO or RTA 405. After 0, 24, 48, and 72 hours cells in the 96-well plates were fixed with 50% TCA and processed for the SRB assay as described above. After 72 hours, cells in the 24-well plates were washed with sterile PBS and harvested in lysis buffer for western blots.

### LSL-Kras^G12D^ murine embryonic fibroblasts

Recombination of the Kras^LSL-G12D^ allele was performed *in vitro* using a self-excising Cre recombinase. MEFs were infected with 500–1000 MOI of mock, Ad-EGFP or Ad-Cre/EGFP (GeneCopoeia, Rockville, MD USA) adenovirus particles according to the manufacturer’s protocol. Cells were harvested 72 hours post-infection. Genomic DNA was isolated using the DNeasy Blood & Tissue Kit (Qiagen) and PCR was performed to evaluate recombination as described (http://web.mit.edu/jacks-lab/protocols/KrasCond_tablesTWO.html). Levels of wild-type and Kras^G12D^ proteins were assessed by western blot.

### Statistical Analyses

All experiments were performed in triplicate, unless otherwise specified. All statistical analyses were performed using GraphPad Prism 6.0 software. The methods used to determine statistical significance are described in each figure legend.

## Results

### Classification of human tumor cell lines by basal NRF2 activity

NRF2 has been reported to be constitutively active in tumors with mutant KEAP1. The main objective of our study was to determine whether NRF2 activation by RTA 405 increases cell growth or survival in human tumor lines. We reasoned that RTA 405 would be unable to increase NRF2 activity in cell lines with low or mutant KEAP1; however, the status of KEAP1 and the basal activity level of NRF2 has been reported for very few common cancer lines [34;48;56]. Therefore, in order to evaluate the effect of RTA 405, we first conducted a series of experiments to characterize the status of KEAP1 and NRF2 in a panel of twenty human tumor lines (Table 1). To accomplish this, we sequenced all coding exons of *KEAP1* and exon 2 of *NFE2L2* to determine whether any of the cell lines had mutations in these genes. We limited *NFE2L2* sequencing to exon 2 because it encodes the KEAP1-interacting domain and has previously been shown to harbor activating mutations [30;53]. Our sequencing results confirmed the previously identified *KEAP1* mutations in the A549 and NCI-H460 non-small cell lung cancer cell lines [54] and identified a novel mutation (Q193H) in the NCI-H23 cell line, which is also derived from non-small cell lung cancer (Table 1). We found no mutations in exon 2 of *NFE2L2* in this panel of cell lines.

**Table 1:** Characteristics of Human Tumor Cell Lines with Different Levels of Basal Nrf2 Activity.

			Relative Protein Levels		
Cell Line	Cancer Type	KEAP1 mutation	Total KEAP1	Nuclear NRF2	Total NQO1
**Low Basal Nrf2 Activity**					
MG-63	Osteosarcoma	-	+++	+/-	-
BxPC-3	Pancreatic adenocarcinoma	-	+++	-	+/-
PANC-1	Pancreatic epithelioid carcinoma	-	+++	-	-
HCT 116	Colorectal carcinoma	-	+++	+	-
MDA-MB-231	Breast adenocarcinoma	-	+++	++	-
786–0	Renal cell adenocarcinoma	-	++	+	-
NCI-H23	Lung (NSCLC)	Q193H	++	+	+/-
SK-N-SH	Neuroblastoma	-	+	+/-	-
**Moderate Basal Nrf2 Activity**					
MCF-7	Breast adenocarcinoma	-	++	-	+++
HT-29	Colorectal adenocarcinoma	-	++	+	+++
G-361	Melanoma	-	++	+	+++
HepG2	Hepatocellular carcinoma	-	++	++	+++
HCT-15	Colorectal adenocarcinoma	-	++	++	+++
**High Basal Nrf2 Activity**					
A2058	Melanoma	-	-	++	+++
SK-MEL-5	Melanoma	-	+/-	++	++++
HeLa	Cervical adenocarcinoma	-	+/-	++	+++
A498	Renal carcinoma	-	+	++++	+
DU 145	Prostate carcinoma	-	-	+++	++++
A549	Lung carcinoma	G333C	-	+++	++++
NCI-H460	Lung carcinoma (Large Cell)	D236H	++	++++	++++

Symbols used to denote relative protein levels: (-) Absent; (+/-) very low; (+) low; (++) moderate; (+++) high; (++++) very high

**Fig 1:**
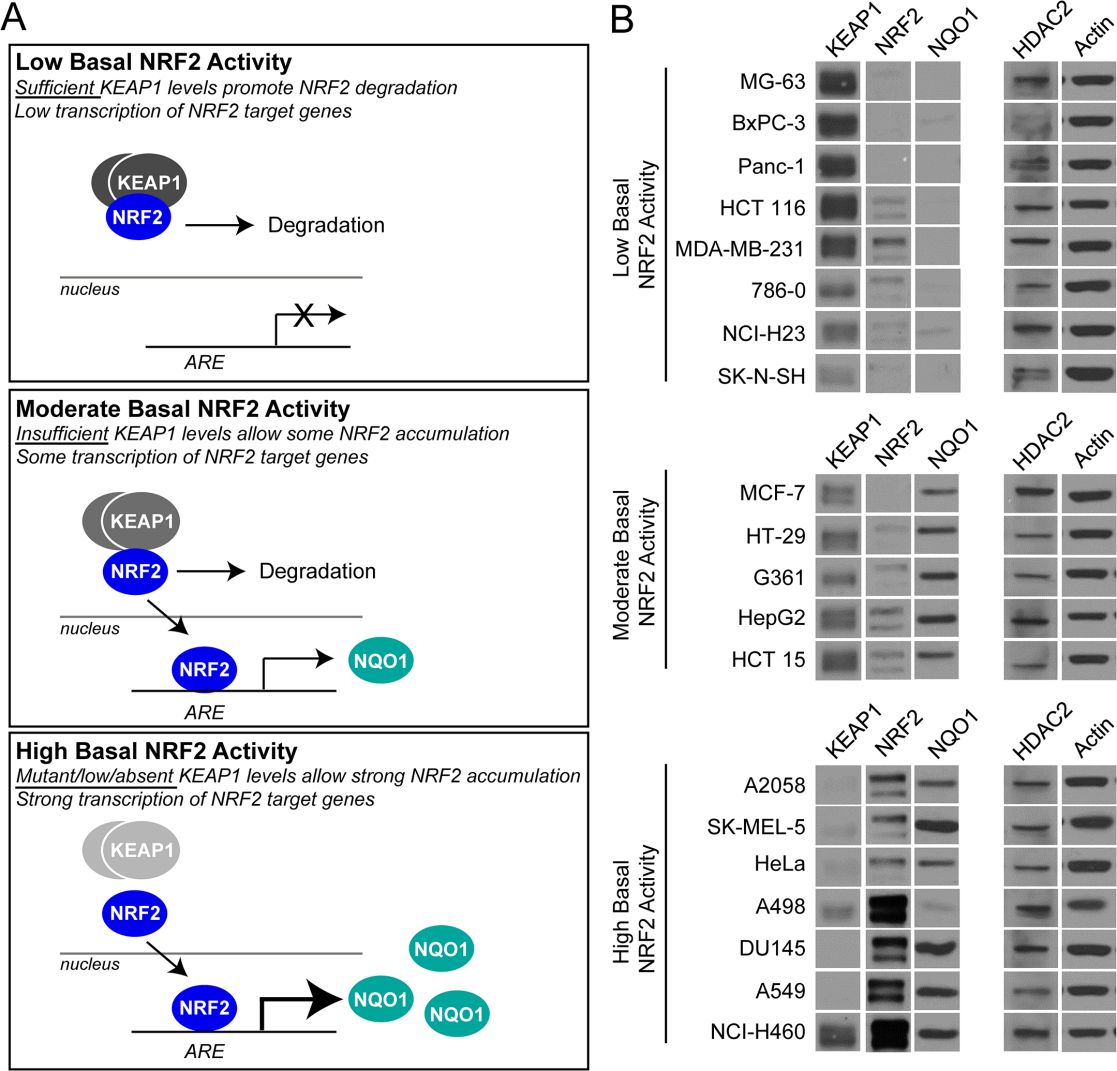
Assessment of basal NRF2 activity in a panel of human tumor cell lines. **A**. Schematic diagram showing characteristics of cell lines with low (upper panel), moderate (middle panel), or high (lower panel) basal NRF2 activity. **B.** Protein levels of KEAP1 and NQO1 (whole-cell lysate), and NRF2 (nuclear fraction), were evaluated by western blot. Actin (whole-cell lysate) and HDAC2 (nuclear fraction) served as loading controls. Based on KEAP1, NRF2, and NQO1 protein levels, cell lines were categorized according to their basal NRF2 activity.

In addition to *KEAP1* and *NFE2L2* mutations, other mechanisms—such as promoter hypermethylation, miRNA expression, and oncogenic signaling—can result in high constitutive levels of NRF2 activity [32–35]. To determine whether any of the cell lines without *KEAP1* or *NFE2L2* mutations had high basal levels of NRF2 activity, we measured KEAP1, nuclear NRF2, and NQO1 (a prototypical NRF2 target gene) protein levels by western blot. We found that, despite having wild-type *KEAP1* and *NFE2L2* genes, several of the cell lines had characteristics of elevated NRF2 activity. To facilitate analysis, we grouped the cell lines into three categories: those with low, moderate, or high basal NRF2 activity (Fig 1A; uncropped blots are in S1 Fig). In cell lines with **low** basal NRF2 activity (*n* = 8), the activity of KEAP1 appeared sufficient to promote NRF2 degradation, resulting in low levels of NQO1 (Fig 1A and 1B, upper panels). In cell lines with **moderate** basal NRF2 activity (*n* = 5), wild-type KEAP1 was detectable, but is appeared to be insufficient to completely suppress NRF2 activity, resulting in detectable levels of NQO1 (Fig 1A and 1B, middle panels). Cell lines with **high** basal NRF2 activity (*n* = 7) had either mutant or low levels of KEAP1, which appeared to render it unable to target NRF2 for degradation (Fig 1A and 1B, lower panels). As a result, high levels of nuclear NRF2 and NQO1 were detected in these cell lines.

The majority of the cell lines could be categorized into one of the three groups; however, a few possessed characteristics of more than one group. For example, when compared to other cell lines in the low basal NRF2 activity group, the SK-N-SH line had relatively low levels of KEAP1 (Fig 1B, upper panel). However, based on the low levels of nuclear NRF2 and NQO1, the KEAP1 levels in this line appeared to be sufficient to promote NRF2 degradation. Conversely, when compared to the other cell lines in the high basal NRF2 activity group, the NCI-H460 line had relatively high levels of KEAP1 (Fig 1B, lower panel). However, it is known that *KEAP1* is mutated (D236H) in NCI-H460 cells and that NRF2 is constitutively active [54]. Despite very high levels of nuclear NRF2 in the A498 cell line, relatively low levels of NQO1 were detected. However, this cell line exhibited several other characteristics that were consistent with high basal NRF2 activity (see below); which suggests that NQO1 itself might be lost or mutated. Finally, although KEAP1 was mutant (Q193H) in the NCI-H23 cell line (Table 1), NRF2 activity appeared to be low, suggesting that the Q193H mutation may be a polymorphism that does not reduce KEAP1 function. This is consistent with results from a recent study where 4 of 18 KEAP1 mutations identified in lung cancer specimens did not impair the ability of KEAP1 to promote NRF2 degradataion [57].

### Characterization of human tumor cell lines with different levels of basal NRF2 activity

**Fig 2:**
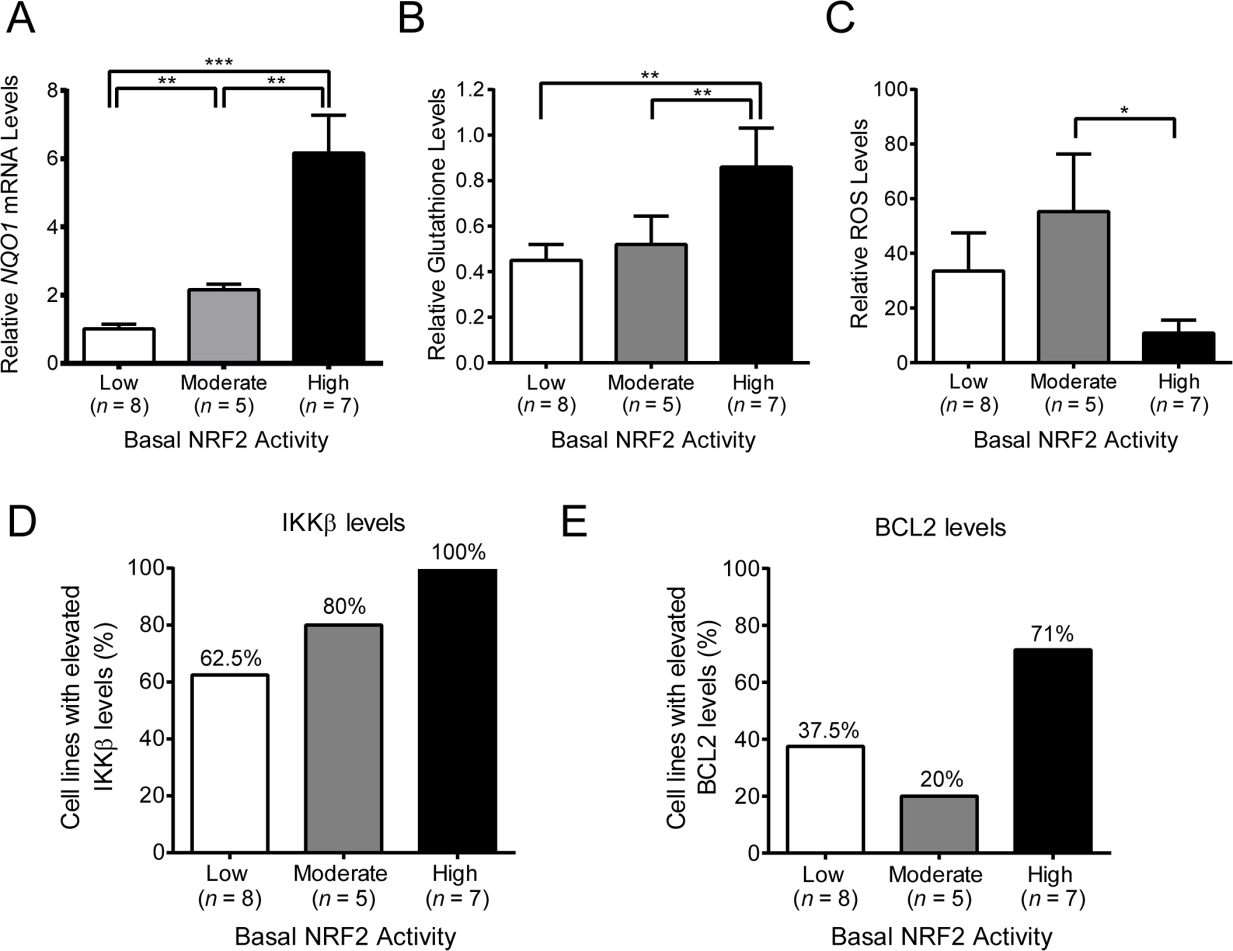
Basal levels of NRF2 and KEAP1 targets in a panel of human tumor cell lines. **A**. *NQO1* mRNA levels for individual cell lines were measured by qPCR and normalized to the average basal *NQO1* level for all cell lines with low basal NRF2 activity. **B**. Total glutathione levels for individual cell lines were normalized to NCI-H460 (set to a value of 1), which was run as a reference in each experiment. Values shown were normalized to the average basal glutathione level for all cell lines with low basal NRF2 activity. **C**. Reactive oxygen species (ROS) levels for individual cell lines were normalized to NCI-H460 (set to a value of 1), which was run as a reference in each experiment. Values shown were normalized to the average basal ROS level for all cell lines with low basal NRF2 activity. **D**-**E**. Basal IKKβ (D) and BCL2 (E) protein levels in human tumor cell lines with low, moderate, or high basal NRF2 activity. Error bars in A-C are SEM. Statistical significance was determined by Mann-Whitney test. *, *P* < .05; **, *P* < .01; ***, *P* < .001.

To further validate the classification of the cell lines, we measured the levels of other biomarkers of NRF2 activity, including *NQO1* mRNA, ROS, and glutathione levels. As mentioned above, *NQO1* is a prototypical NRF2 target gene and one would expect its transcription to be elevated in cell lines with high basal NRF2 activity. When compared to cell lines with low basal NRF2 activity, those with moderate and high basal NRF2 activity had successively higher levels of NQO1 expression (Fig 2A and S2 Fig). In addition to *NQO1*, NRF2 also regulates the expression of several genes involved in glutathione synthesis and increases cellular glutathione levels [58]. Consistent with this, total glutathione levels were significantly elevated in cell lines with high basal NRF2 activity compared to those with low or moderate basal NRF2 activity (Fig 2B and S2 Fig). By increasing glutathione levels, as well as the expression of other antioxidant genes, NRF2 reduces oxidative stress. To assess the level of oxidative stress in each tumor cell line, we measured ROS levels using a fluorescent probe. We found that ROS levels were lowest in the cell lines with high basal NRF2 activity (Fig 2C and S2 Fig). Therefore, in summary, cell lines with high basal NRF2 activity had high NQO1 mRNA levels, high glutathione levels, and low ROS levels. Furthermore, NQO1 and glutathione levels increased in proportion to the level of basal NRF2 activity. In contrast, ROS levels did not follow a similar trend: there was no difference between cell lines with low and moderate NRF2 activity levels. This suggests that moderate activation of NRF2 is sufficient to increase target gene expression and glutathione synthesis, but NRF2 activity must reach a certain threshold, which may only be achieved when KEAP1 is absent or inactive, in order to reduce ROS levels.

In addition to NRF2, KEAP1 regulates the levels of other proteins, including IKKβ and BCL2 [59;60]. KEAP1 directly binds to, and facilitates the ubiquitination of IKKβ and BCL2 by the CUL3/RBX1 E3 ligase complex. This leads to degradation of IKKβ and BCL2 by the proteasome. Accordingly, loss of KEAP1 in cell lines results in elevated levels of IKKβ and BCL2 [59;60], and IKKβ levels are elevated in human tumors with low KEAP1/CUL3 levels [59;61]. IKKβ is a kinase that promotes degradation of nuclear factor of kappa light polypeptide gene enhancer in B-cells inhibitor, alpha (NFKBIA or IκBα), a suppressor of the NF-κB transcription factor which controls the expression of many genes involved in survival, and BCL2 is an oncogene with anti-apoptotic activity. Given the relationship between KEAP1, IKKβ, BCL2, and cancer, we investigated whether the tumor cell lines with high basal NRF2 activity also had elevated IKKβ and BCL2 protein levels. We found that many cell lines in the panel tended to have high levels of IKKβ (S3 Fig). However, IKKβ levels were elevated in 100% of cell lines with high basal NRF2 activity, compared with 80% of the cell lines with moderate NRF2 activity, and 62.5% of the cell lines with low NRF2 activity (Fig 2D). When we investigated BCL2, we found that fewer of the cell lines had elevated BCL2 levels (S3 Fig). In the cell lines with high basal NRF2 activity, 71% also had high BCL2 levels, compared with 37.5% and 20% of the cell lines with low and moderate NRF2 activity, respectively. Like glutathione levels, IKKβ levels increased in proportion to the levels of basal NRF2 activity, whereas BCL2 levels were elevated in a larger fraction of cell lines that had high NRF2 activity, as compared to those with low or moderate NRF2 activity. This suggests that different KEAP1 substrates may have different sensitivities to changes in KEAP1 function. Taken together, these data are consistent with the notion that loss of KEAP1 affects multiple targets that are relevant to cancer cell biology, including not only NRF2, but also IKKβ and BCL2 [62].

### IKKβ and BCL2 levels in *Keap1* ^-/-^ murine embryonic fibroblasts

**Fig 3:**
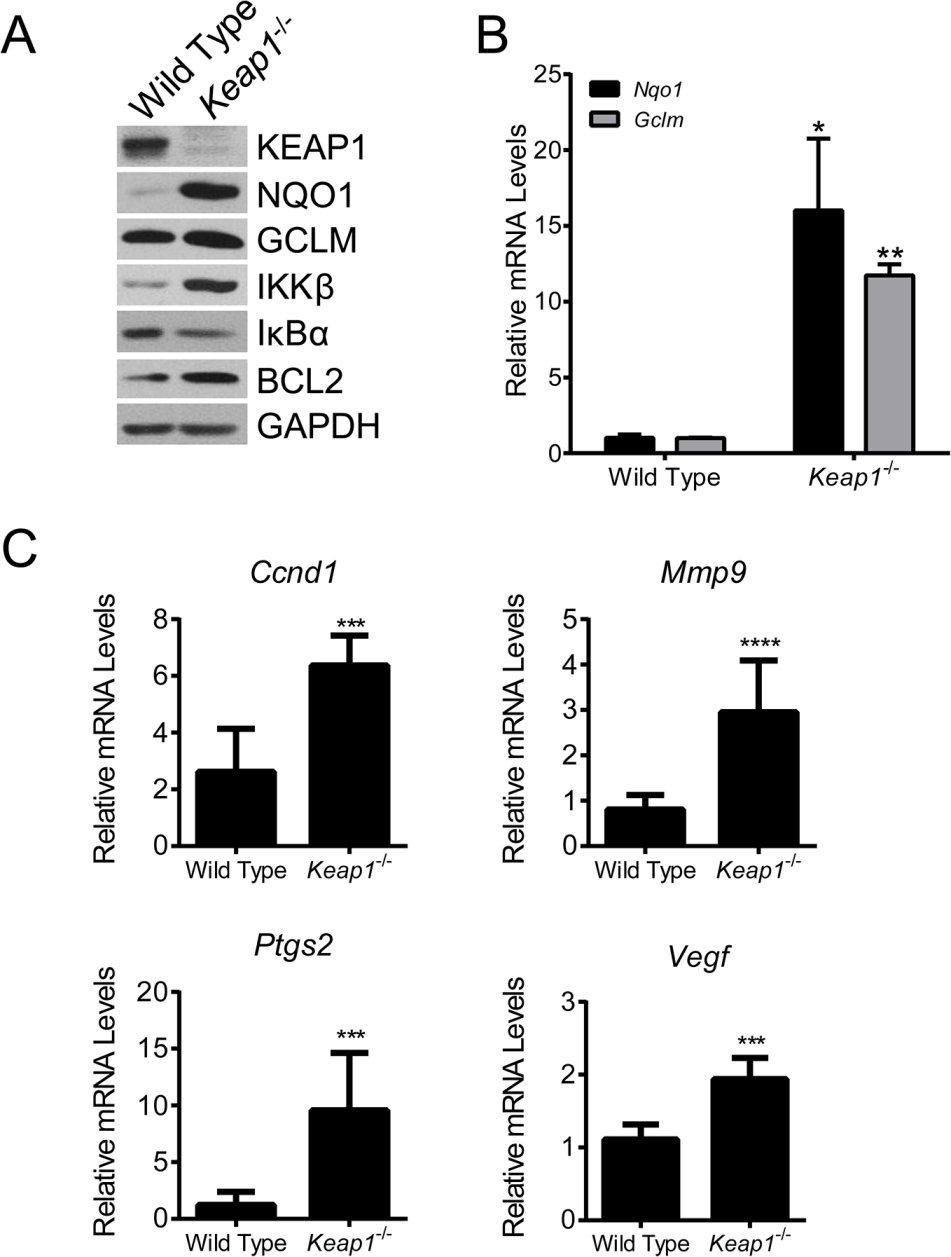
IKKβ and BCL2 levels in *Keap1* ^-/-^ murine embryonic fibroblasts. **A**. Basal levels of KEAP1-interacting proteins and downstream targets were evaluated in WT and *Keap1* ^-/-^ MEFs by western blot. GAPDH served as a loading control. **B.** Basal mRNA levels of *Nqo1* and *Gclm* in WT and *Keap1* ^-/-^ MEFs measured by qPCR. mRNA levels in *Keap1* ^-/-^ cells were normalized to WT cells. *, *P* < .05; **, *P* < .01 vs. WT by paired t-test. **C**. Basal mRNA levels of *Ccnd1*, *Mmp9*, *Ptgs2*, and *Vegf* in WT and *Keap1* ^-/-^ MEFs measured by qPCR. mRNA levels in *Keap1* ^-/-^ cells were normalized to WT cells. ***, *P* < .001; ****, *P* < .0001 vs. WT by t-test. For all panels, data points are the mean of three independent experiments. Error bars are SD.

We have shown that the levels of IKKβ and BCL2 tend to be higher in tumor cell lines that have high basal NRF2 activity, suggesting that mutant or low/absent levels of KEAP1 may contribute to elevated IKKβ and BCL2 levels. We next used wild-type (WT) and *Keap1* ^-/-^ murine embryonic fibroblasts (MEFs) as a model to directly assess the relationship between KEAP1, IKKβ, and BCL2 levels. As previously reported [49], NRF2 was elevated in *Keap1* ^-/-^ MEFs (S4 Fig). Treatment with RTA 405 increased NRF2 levels in WT MEFs, but not in *Keap1* ^-/-^ MEFs (S4 Fig). Consistent with NRF2 activation, the protein (A) and mRNA (Fig 3B) levels of NQO1 and GCLM, two NRF2 target genes, were elevated in *Keap1* ^*-/-*^ MEFs. In addition to elevated levels of NRF2 and its downstream target genes, we also found that the levels of IKKβ and BCL2 were higher in *Keap1* ^-/-^ MEFs than in WT MEFs (Fig 3A). To determine whether the elevated IKKβ levels correlated with an increase in its activity, we assessed the levels of downstream components of the NF-κB signaling pathway. IKKβ phosphorylates IκBα, which leads to its degradation and subsequent activation of the NF-κB transcription factor. Consistent with higher IKKβ activity, we observed lower levels of IκBα (Fig 3A) and significantly higher expression levels of several NF-κB target genes, including *Ccnd1*, *Mmp9*, *Ptgs2*, *Vegf*, *and Bcl2l1*, in *Keap1* ^-/-^ MEFs as compared to WT MEFs (Fig 3C and S5 Fig). There were no significant differences in *Ccl2*, *Birc3* or *Il1b* levels, and for unknown reasons *Ccl5* levels were significantly higher in WT MEFs than in *Keap1* ^-/-^ MEFs (S5 Fig). Taken together, these results demonstrate that loss of KEAP1 results in higher IKKβ and BCL2 levels, and that the elevated level of IKKβ leads to an increase in NF-κB transcriptional activity. These results are consistent with the observation that loss of KEAP1 was correlated with higher NF-κB transcriptional activity in human tumors [61].

### Growth rate of cells with different levels of basal NRF2 activity

**Fig 4:**
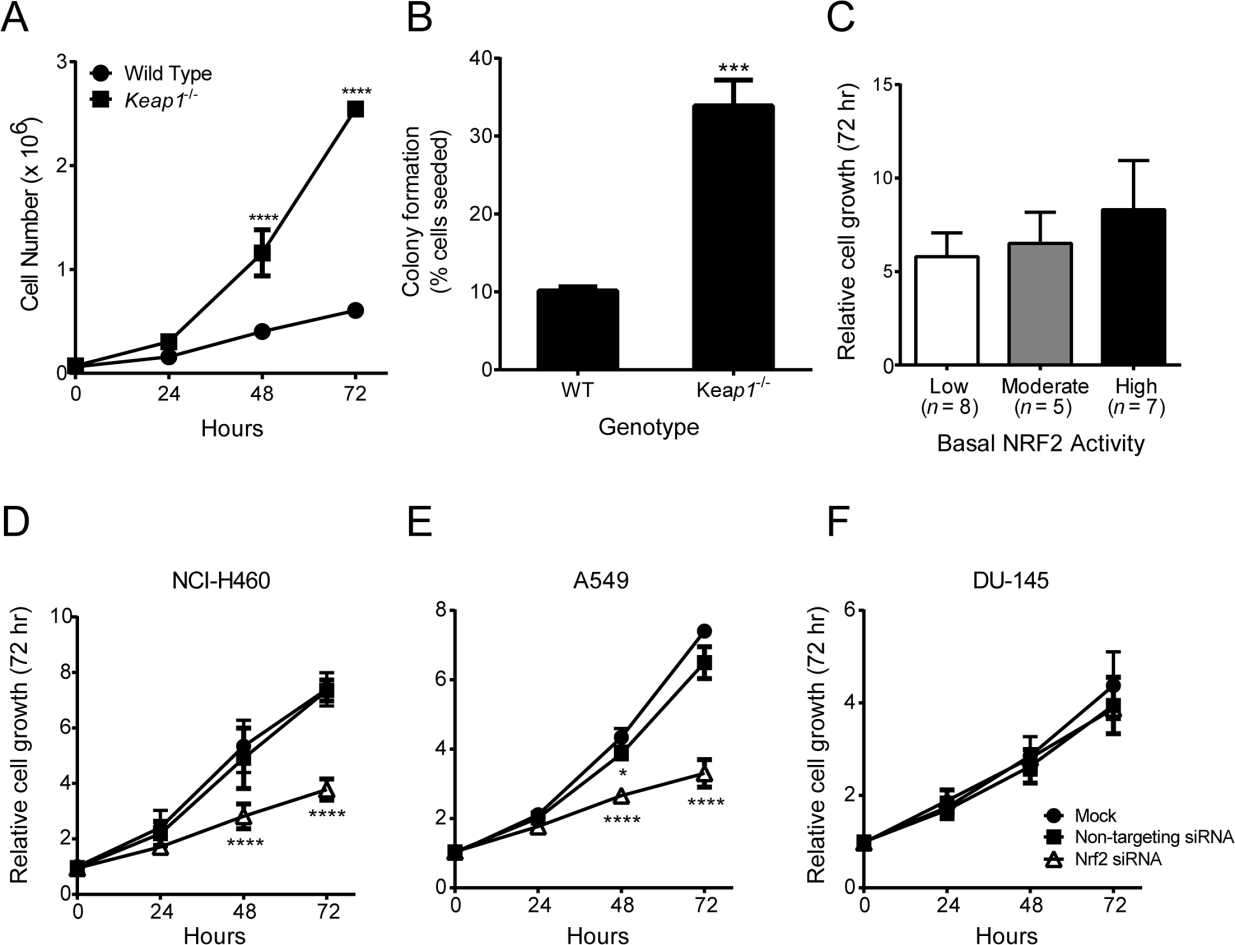
Growth rate of cells with different levels of basal NRF2 activity. **A**. Growth of WT and *Keap1* ^-/-^ MEFs over a 72-hour period. ****, *P* < .0001 vs. WT by t-test. **B**. Colonies formed by WT and *Keap1* ^-/-^ MEFs. Percentage of seeded cells that formed colonies is shown. ***, *P* < .001 vs. WT by t-test. **C**. Growth of human tumor cell lines with low, moderate, or high basal NRF2 activity over a 72-hour period. Relative growth was determined by dividing SRB absorbance at 72 hours by SRB absorbance at 0 hours. **D-F**. Effect of NRF2 siRNA on growth of human tumor cell lines. Growth was assessed in NCI-H460 (D), A549 (E), and DU 145 (F) cells using the SRB assay 24, 48, and 72 hours after transfection with non-targeting siRNA or NRF2 siRNA. Mock transfected cells served as a control. *, *P* < .05; ****, *P* < .0001 vs. Mock by repeated measures two-way ANOVA and Dunnett’s multiple comparison test. For all panels, data points are the mean of three independent experiments. Error bars in (C) are SEM. All other error bars are SD.

Loss of KEAP1 has been reported to increase the proliferation rate of MEFs [48;63]. To confirm and expand these studies, we counted the number of viable WT and *Keap1* ^-/-^ MEFs at 24-hour intervals after seeding. We found that significantly more *Keap1* ^-/-^ MEFs than WT MEFs were present at 48 and 72 hours (Fig 4A). In addition, when the MEFs were seeded at low density, a higher percentage of colonies were formed by *Keap1* ^-/-^ MEFs than by WT MEFs (Fig 4B). These data indicate that loss of KEAP1, which results in elevated levels of NRF2, IKKβ, and BCL2, confers a growth and survival advantage to MEFs. To determine whether loss of functional KEAP1 is also correlated with increased proliferation in human tumor cells, we used the sulforhodamine B (SRB) assay to assess the growth of each of the 20 cell lines over a 72-hour period. In contrast to the loss of KEAP1 in MEFs, loss of functional KEAP1 and high basal NRF2 activity in human tumor cell lines was not correlated with a significant increase in growth (Fig 4C and S6 Fig). This result was not entirely unexpected given that tumor cell proliferation is likely influenced by other dysregulated oncogenic signaling pathways. Although the growth of cell lines with high basal NRF2 activity was not significantly different than those with moderate or low basal NRF2 activity, elevated NRF2 activity could still be contributing to the growth of individual cell lines. To assess this, we used siRNA to reduce NRF2 levels in three cell lines with high basal NRF2 activity: A549, NCI-H460, and DU 145 (S7 Fig). Consistent with previously published results [64], A549 and NCI-H460 cells that were transfected with NRF2 siRNA grew at a slower rate than those that were mock transfected or transfected with non-targeting siRNA (Fig 4D and 4E). Therefore, in these two cell lines, high NRF2 activity appears to be required for optimal growth. In contrast, the growth of DU 145 prostate cancer cells was not affected by NRF2 siRNA (Fig 4F). These results suggest that the degree of reliance on high NRF2 activity for growth and survival may be greater for some tumor cell lines than for others.

### RTA 405 differentially affects the stability of KEAP1 target proteins

We have demonstrated that loss of KEAP1 in MEFs increased NRF2, IKKβ, and BCL2 levels, as well as the rate of proliferation. Similarly, human tumor cell lines with high basal NRF2 activity also tend to have elevated IKKβ and BCL2 (Fig 2D and 2E). In the clinic, tumors with this profile have been reported to be more aggressive and resistant to therapy [30;31;61]. As described above, it is well-established that the AIMs bind to KEAP1 and block its ability to target NRF2 for degradation [8]. However, the effect of AIMs on *other* KEAP1 target proteins has not previously been assessed. Therefore, we treated WT and *Keap1* ^-/-^ MEFs, as well as the panel of 20 human tumor lines, with RTA 405 and measured the protein levels and activities of NRF2, BCL2 and IKKβ.

**Fig 5:**
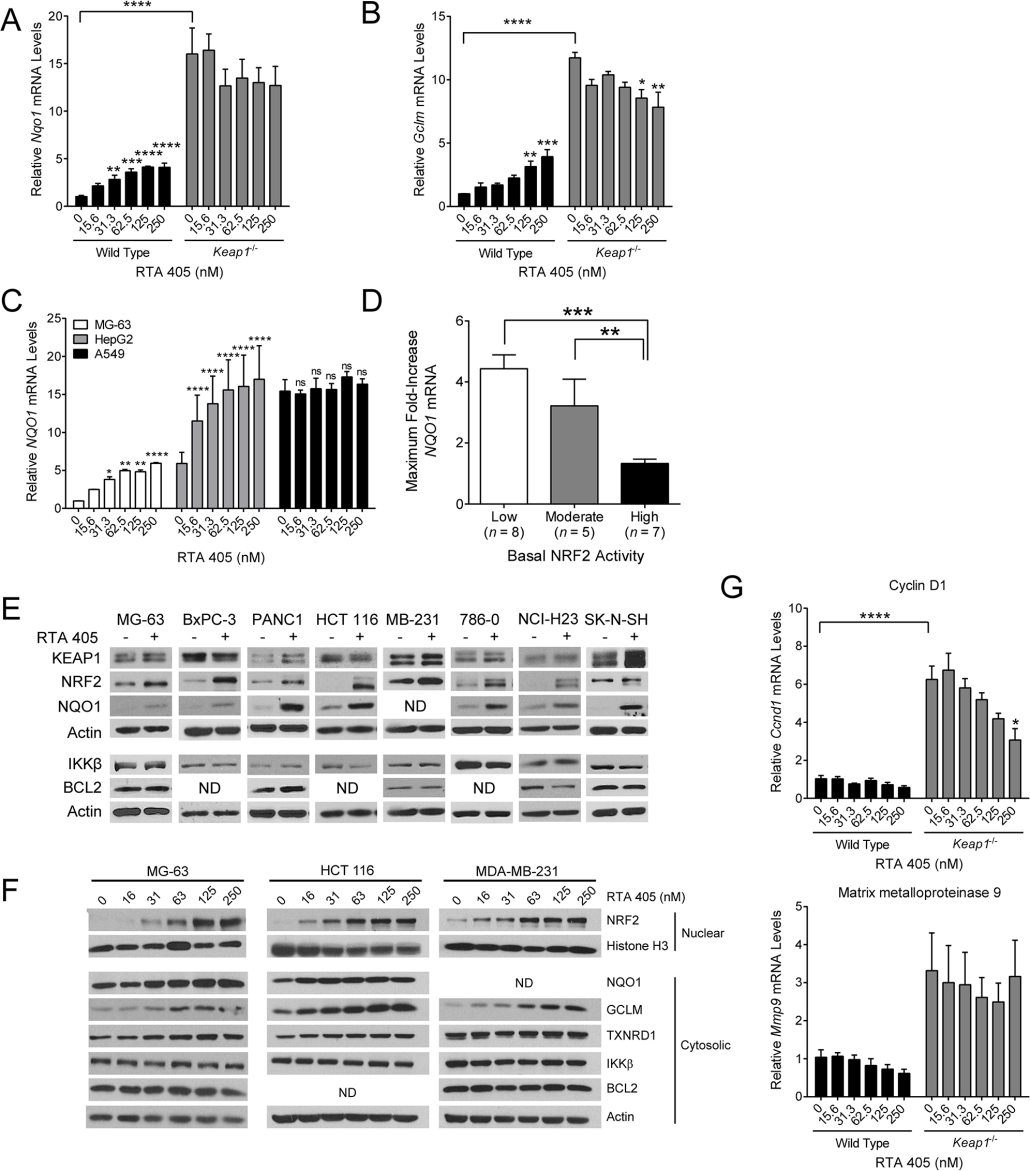
Effect of RTA 405 treatment on the levels and activities of NRF2, IKKβ, and BCL2. **A-B.** Effect of 18-hour RTA 405 treatment on mRNA levels of *Nqo1* (A) and *Gclm* (B) assessed by qPCR in MEFs. mRNA levels were normalized to vehicle-treated WT cells. Data points are the mean of three experiments. Error bars are SEM. Statistical significance was determined by one-way ANOVA and Dunnett’s multiple comparisons test. **, *P* < 0.01; ****, *P* < 0.0001. **C**. MG-63, Hep-G2, and A549 cells were treated with the indicated concentrations of RTA 405 for 18 hours and *NQO1* mRNA levels were assessed by qPCR. *NQO1* mRNA levels were normalized to vehicle-treated MG-63 cells. Data points are mean of three experiments. Error bars are SD. Statistical significance was determined by one-way ANOVA and Dunnett’s multiple comparisons test. Comparisons were made between each concentration of RTA 405 and the vehicle control for each cell line. *, *P* < 0.05; **, *P* < 0.01; ****, *P* < 0.0001; ns, not significant. **D**. Mean maximum observed fold-increase in *NQO1* mRNA levels following treatment with RTA 405 for 18 hours. Error bars are SEM. Statistical significance was determined by Mann-Whitney test. **, *P* < 0.01; ***, *P* < 0.001. **E**. Protein levels of KEAP1-interacting proteins and downstream targets were evaluated by western blot in 8 human tumor cell lines with low basal NRF2 activity following treatment with vehicle or 250 nM RTA 405 for 24 hours. Actin served as a loading control. ND, none detected. **F**. Protein levels of NRF2 (nuclear fraction) and NQO1, GCLM, TXNRD1, IKKβ, and BCL2 (cytosolic fraction) were evaluated by western blot in the MG-63 osteosarcoma, HCT 116 colon carcinoma, and MDA-MB-231 mammary carcinoma cell lines following treatment with vehicle or RTA 405 for 24 hours. Actin (whole-cell lysate) and Histone H3 (nuclear fraction) served as loading controls. **G**. Effect of 18-hour RTA 405 treatment on mRNA levels of *Ccnd1* (top panel) and *Mmp9* (bottom panel) assessed by qPCR. mRNA levels were normalized to those in vehicle-treated WT cells. Data points are the mean of three independent experiments. Error bars are SEM. Statistical significance was determined by one-way ANOVA and Dunnett’s multiple comparisons test. *, *P* < 0.05; ****, *P* < 0.0001.

In WT MEFs, RTA 405 treatment dose-dependently increased *Nqo1* and *Gclm* mRNA levels, but did not further increase the already elevated mRNA levels in *Keap1* ^-/-^ MEFs (Fig 5A and 5B). This is consistent with the established mechanism of action of the AIMs—direct binding to KEAP1 C151 blocks NRF2 degradation and results in its accumulation and activation as a transcription factor [5]. It is notable that the maximal induction of *Nqo1* and *Gclm* by RTA 405 in WT MEFs was much lower than the basal levels of these NRF2 targets in *Keap1* ^-/-^ MEFs. In the tumor cell lines, we found that the magnitude of NRF2 target gene induction by RTA 405 was inversely correlated with the basal level of NRF2 activity (Fig 5C and 5D). RTA 405 dose-dependently increased *NQO1* mRNA levels in cell lines with low basal NRF2 activity (Fig 5C, MG-63). We also observed a dose-dependent increase in *NQO1* mRNA levels in cells with moderate basal NRF2 activity, but the magnitude (fold) of the increase was lower (Fig 5C, HepG2). Similar to *Keap1* ^-/-^ MEFs, cell lines with high basal NRF2 activity exhibited little or no increase in *NQO1* mRNA levels following RTA 405 treatment (Fig 5C, A549). We observed similar trends for the other cell lines in each group (S8 Fig). The average maximal RTA 405-mediated increase in *NQO1* mRNA levels was significantly lower in cell lines with high basal NRF2 activity than in cell lines with low or moderate basal NRF2 activity (Fig 5D). We also observed similar trends in *NQO1* induction in tumor cells treated with another AIM, bardoxolone methyl (data not shown).

We next asked whether RTA 405 treatment increases the levels of IKKβ and BCL2 in tumor cells. In cells with low basal NRF2 activity, treatment with 250 nM RTA 405 increased the levels of NRF2 and NQO1; however, RTA 405 did not increase the levels of IKKβ or BCL2 (Fig 5E). RTA 405 also did not increase IKKβ or BCL2 levels in most cells with moderate or high basal NRF2 activity (S9 Fig). We did observe an increase in BCL2 levels in one cell line with moderate basal NRF2 activity (HCT-15 line; S9 Fig), but the underlying mechanism is not known. To further examine the effect of RTA 405 on IKKβ and BCL2, we treated three cell lines that had low basal NRF2 activity with RTA 405 concentrations ranging from 15 to 250 nM. We observed concentration-dependent increases in nuclear NRF2 and several NRF2 targets; however in clear contrast, we did not observe an increase in IKKβ or BCL2 levels following treatment with RTA 405 (Fig 5F). These results indicate that RTA 405 modulates KEAP1 activity in a way that appears to increase the stability of NRF2, but not IKKβ or BCL2. AIMs have been shown to inhibit NF-κB signaling in a variety of contexts [2;27;28]. Consistent with this, RTA 405 treatment decreased the mRNA levels of NF-κB target genes, *Ccnd1* and *Mmp9*, in WT MEFs (Fig 5G). Notably, RTA 405 also reduced the elevated basal *Ccnd1* mRNA levels in *Keap1* ^-/-^ MEFs, suggesting that RTA 405 could counteract the effects of KEAP1 loss on the NF-κB signaling pathway.

### RTA 405 inhibits growth and survival equivalently in cells with different basal NRF2 activity

**Fig 6:**
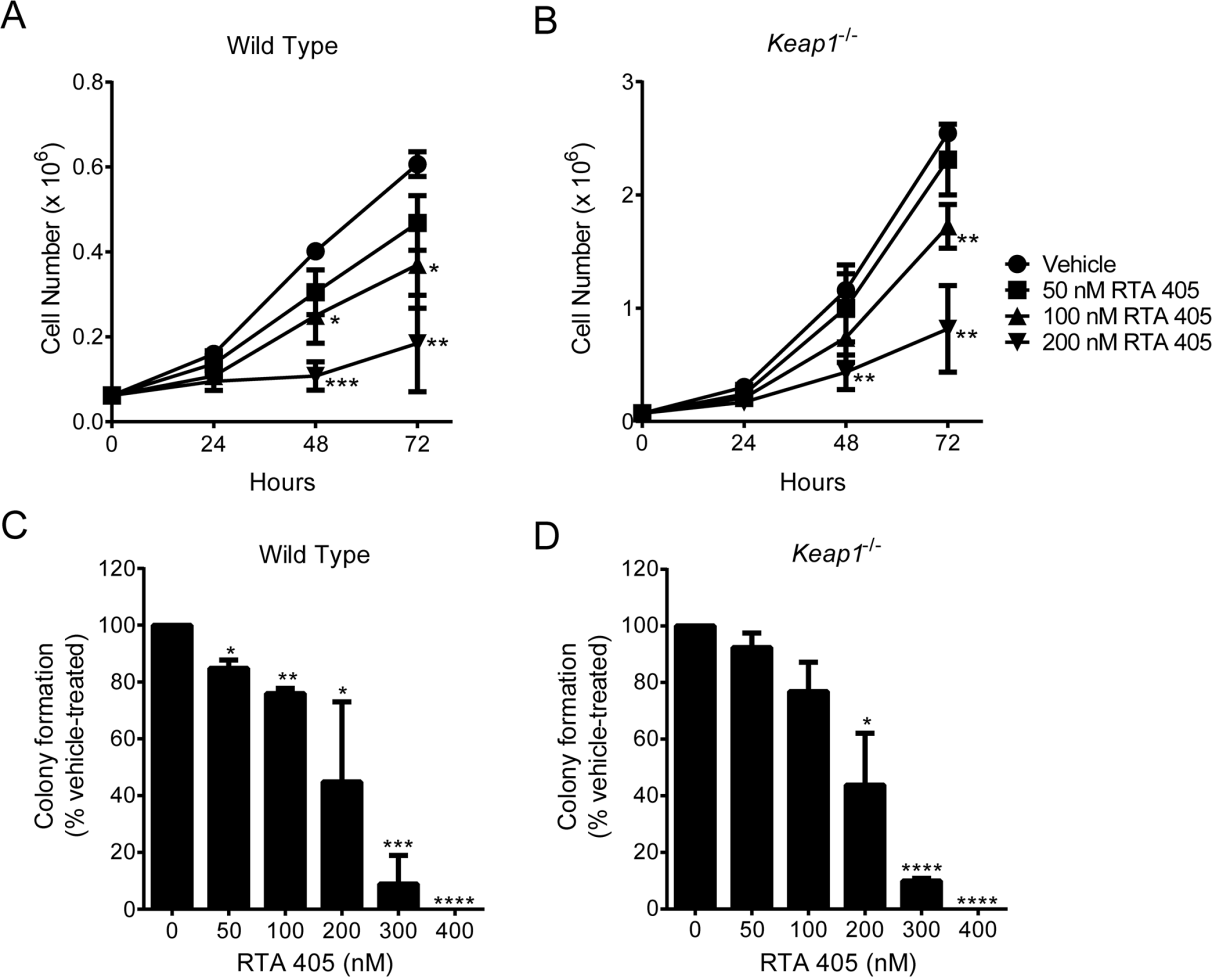
RTA 405 inhibits growth and colony formation in wild type and *Keap1* ^-/-^ murine embryonic fibroblasts. **A-B.** Effect of RTA 405 on growth of WT (A) and *Keap1* ^-/-^ (B) MEFs over a 72-hour period. **C-D.** Effect of RTA 405 on colony formation in WT (C) and *Keap1* ^-/-^ (D) MEFs. In all panels, data points are the mean of three independent experiments and error bars are SD. Statistical significance was determined by t-test. *, *P* < 0.05; **, *P* <0.01; ***, *P* < 0.001; ****, *P* < 0.0001 vs. vehicle-treated cells.

The increase in proliferation and survival observed in MEFs derived from *Keap1* ^*-/-*^ *mice* has been proposed to be the result of high NRF2 activity [30;31]. Since RTA 405 increases NRF2 activity in MEFs, we asked whether RTA 405 treatment would also increase MEF proliferation. Despite clear activation of NRF2 in WT MEFs (Fig 5A), we found that RTA 405 **inhibited** growth and colony formation (Fig 6A and 6C). Treatment with RTA 402 (bardoxolone methyl) produced similar results (S10 Fig). These results demonstrate that treatment with AIMs and loss of KEAP1 do not have the same effects on MEF proliferation and survival. RTA 405 also inhibited growth and colony formation in *Keap1* ^-/-^ MEFs (Fig 6B and 6D), demonstrating that RTA 405 is able to counteract the effects of KEAP1 loss on cell proliferation and survival.

**Fig 7:**
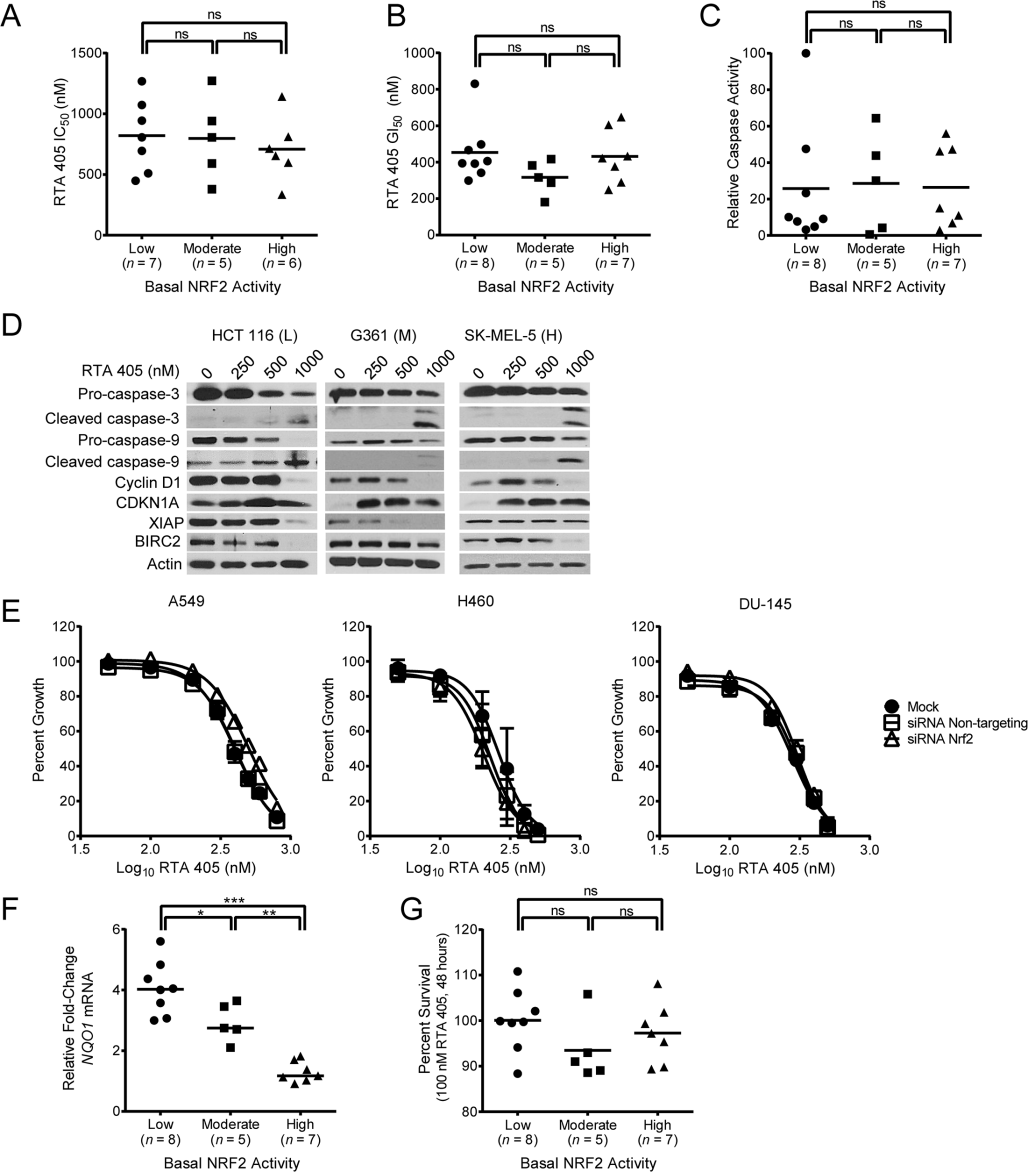
Effect of RTA 405 on survival in cell lines with low, moderate, or high basal NRF2 activity. **A**. IC_50_ values for cell lines treated with RTA 405 for 48 hours. IC_50_ values for 2 cell lines (SK-MEL-5 and SK-N-SH) could not be determined using the tested concentration range and are excluded from the graph. **B**. GI_50_ values for cell lines treated with RTA 405 for 72 hours. **C**. Maximum RTA 405-induced caspase-3/7 activity observed in cells treated with 1600 nM RTA 405 for 24 hours. Caspase-3/7 activity was normalized to activity in 786–0 cells (value, 100). **D**. Cells with low (L), moderate (M), or high (H) basal NRF2 activity were treated with RTA 405 for 24 hours and levels of caspase-3, caspase-9, cyclin D1, CDKN1A (p21), XIAP, and BIRC2 were evaluated by western blot in whole-cell lysates. Actin served as a loading control. **E**. Cells were mock transfected or transfected with non-targeting or NRF2 siRNA and then treated with RTA 405 for 72 hours. Growth was assessed using the SRB assay. **F**. Effect of 125 nM RTA 405 on *NQO1* mRNA levels. Cells were treated with RTA 405 for 18 hours and *NQO1* mRNA levels were assessed by qPCR. **G**. Cells were treated with 100 nM RTA 405 for 48 hours and cell viability was determined. Percent of vehicle-treated cell survival is shown. For (A, B, G) cell viability was determined using the SRB assay. For (A-C, F, G) data points for individual cell lines are the mean of three individual experiments and horizontal lines are the mean of all cell lines in each group. Statistical significance was determined by Mann-Whitney test. ns, not significant; *, *P* < 0.05; **, *P* < 0.01; ***, *P* < 0.001.

It has previously been reported that tumor cell lines that have high NRF2 activity are resistant to anticancer agents [36]. AIMs are known to directly inhibit tumor cell growth and induce apoptosis in an NRF2-independent manner [1]. In this regard, AIMs have been shown to reduce cyclin D1 levels [23], increase p21 levels [23], and increase caspase cleavage [65]. To determine whether the direct anticancer activity of RTA 405 is reduced in tumor cell lines with high NRF2 activity, we treated the panel of 20 tumor cell lines with RTA 405 ranging in concentration from 50 nM to 1000 nM and measured cell viability. We found no significant difference in the mean IC_50_ (Fig 7A) or GI_50_ (Fig 7B) values in cell lines with low, moderate, or high basal NRF2 activity, suggesting that high basal NRF2 levels **do not** provide resistance to RTA 405-mediated growth inhibition. We observed similar results in a subset of cell lines treated with bardoxolone methyl (S10 Fig). In addition, we observed no significant difference in mean caspase-3/7 activity between the three groups of cell lines following treatment with RTA 405 (Fig 7C). Individual values for IC_50_, GI_50,_ and caspase-3/7 activity are in S3 Table. To evaluate the effects of RTA 405 on proteins involved in apoptosis and cell cycle control, we treated the tumor cells with 250 nM, 500 nM, and 1000 nM RTA 405 and assessed protein levels by western blot (Fig 7D and S11 Fig). RTA 405 treatment increased caspase-3 and caspase-9 cleavage in cell lines with low, moderate, or high basal NRF2 activity (Fig 7D, S4 Table, and S11 Fig). Reductions in CCND1, XIAP, and BIRC2 protein levels occurred at similar concentrations. RTA 405 also increased CDKN1A (p21) levels at concentrations ranging from 250 to 1000 nM.

The results above indicate that high basal NRF2 activity levels, reflective of KEAP1 loss or mutation, do not protect tumor cells from RTA 405-mediated growth inhibition. To directly test this, we asked whether reducing NRF2 in cell lines with high basal NRF2 activity would sensitize the tumor cells to RTA 405-mediated growth inhibition. NRF2 siRNA reduced the basal levels of NRF2, NQO1, and GCLM in DU 145, A549, and NCI-H460 cell lines (S7 Fig). However, reducing NRF2 levels had no significant effect on RTA 405-mediated growth inhibition in NCI-H460 or DU-145 cells (Fig 7E). Furthermore, rather than sensitizing A549 cells to RTA 405, NRF2 siRNA resulted in a slight decrease in the sensitivity of these cells to RTA 405-mediated growth inhibition (Fig 7E). These results support the conclusion that high basal NRF2 activity in tumor cells does not provide resistance to RTA 405-mediated growth inhibition.

As shown above, the direct effects of RTA 405 on tumor cell growth are appreciable at concentrations ≥ 250 nM. However, we have shown that RTA 405 increases NRF2 activity at concentrations as low as 16 nM (Fig 5). To assess the effects of low RTA 405 concentrations, we measured *NQO1* mRNA levels and survival in cell lines treated with 100 to 125 nM RTA 405—doses of RTA 405 that increase NRF2 activity, but do not directly reduce growth of tumor cells. As expected, 125 nM RTA 405 increased *NQO1* mRNA levels in cell lines with low and moderate basal NRF2 activity levels, but not in those with high basal NRF2 activity levels (Fig 7F). In contrast, the mean percent survival following treatment with 100 nM RTA 405 was not significantly different between cell lines with low, moderate, or high basal NRF2 activity (Fig 7G). We observed similar results in a subset of cell lines treated with bardoxolone methyl (S10 Fig). Therefore, activation of NRF2 by low concentrations of RTA 405 did not correlate with an increase in tumor cell survival.

### RTA 405 inhibits growth equivalently in cell lines with wild-type or mutant *KRAS*

**Fig 8:**
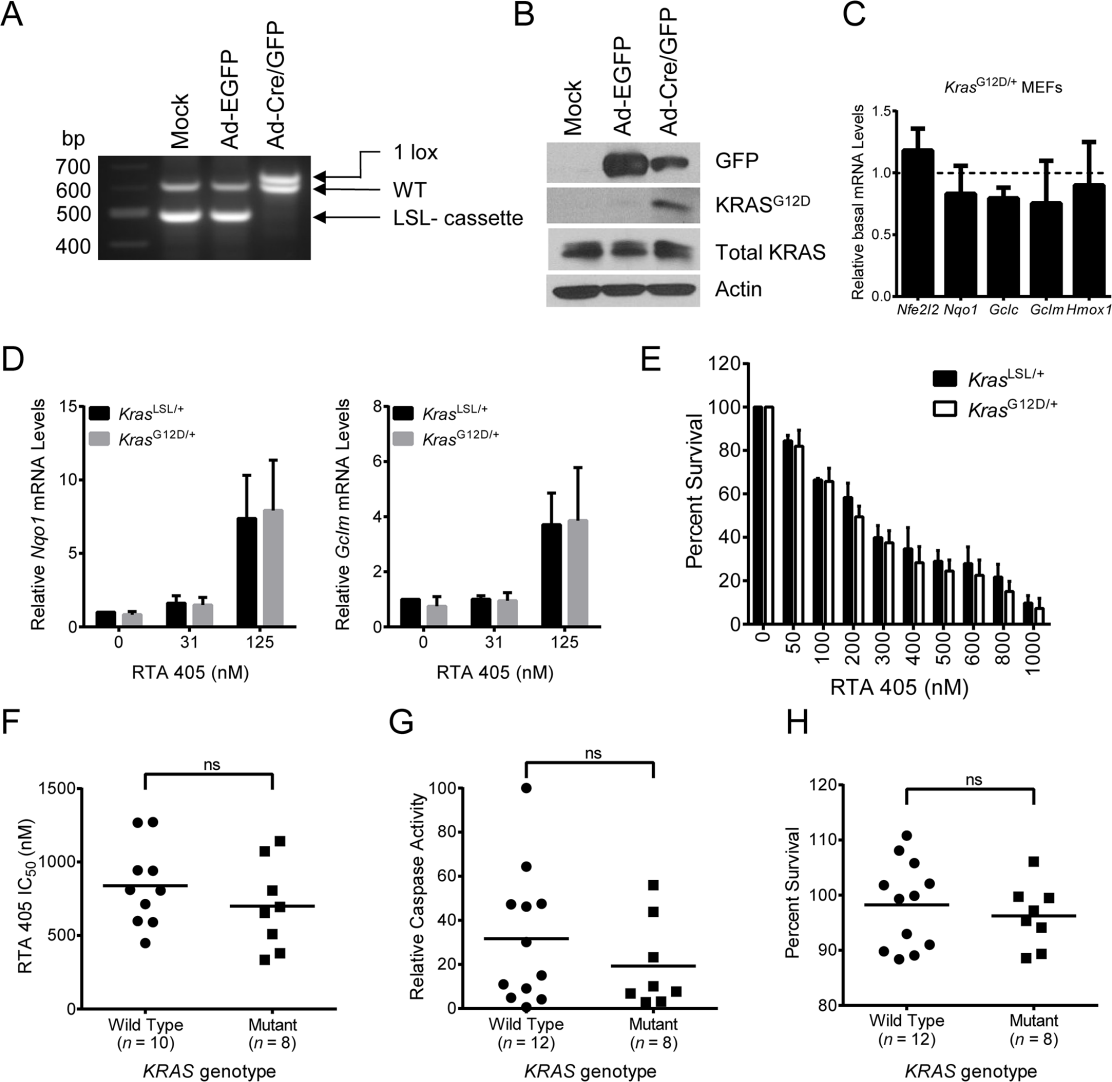
Effect of RTA 405 on survival of WT and *KRAS* mutant cell lines. **A-B.** LSL-*Kras* ^G12D^ MEFs were mock-infected or infected with adenoviral-Cre or adenoviral-EGFP for 72 hours. **A**. PCR was performed using genomic DNA to assess recombination efficiency and excision of the STOP cassette. **B**. Levels of total Kras and Kras^G12D^ proteins were assessed by western blot. GFP and actin served as controls. **C**. Basal mRNA levels of *Nrf2* and NRF2 target genes in Cre-infected LSL-Kras^G12D/+^ MEFs. Target gene mRNA levels in Cre-infected LSL-Kras^G12D/+^ MEFs were normalized to those in mock-infected LSL- Kras^G12D/+^ cells. **D**. Effect of 18-hour RTA 405 treatment on mRNA levels of *Nqo1* (left panel) and *Gclm* (right panel) assessed by qPCR in mock-infected and Cre-infected LSL-*Kras* ^G12D/+^ MEFs. mRNA levels were normalized to vehicle-treated, mock-infected LSL-*Kras* ^G12D^/+ cells. **E**. Percent survival of mock-infected and Cre-infected LSL-*Kras* ^G12D/+^ MEFs treated with RTA 405. Cell viability was determined using the SRB assay 48 hours after treatment. Percent of vehicle-treated cell survival is shown. For (C-E), data points are the mean of three experiments and error bars are SD. **F**. IC_50_ values for cell lines treated with RTA 405 for 48 hours. IC_50_ values for 2 cell lines (SK-MEL-5 and SK-N-SH) could not be determined using the tested concentration range and are excluded from the graph. **G**. Maximum RTA 405-induced caspase-3/7 activity observed in cells treated with 1600 nM RTA 405 for 24 hours. Caspase-3/7 activity was normalized to activity in 786–0 cells (value, 100). **H**. Cells were treated with 100 nM RTA 405 for 48 hours and cell viability was determined. Percent of vehicle-treated cell survival is shown. For (F-H) data points for individual cell lines are the mean of three individual experiments and horizontal lines are the mean of all cell lines in each group. Statistical significance was determined by the Mann-Whitney test. ns, not significant.

Activated KRAS (G12D) has been reported to increase transcription of *NFE2L2* and some NRF2 target genes [35]. Since ablation of *Nfe2l2* reduced KRAS^G12D^-mediated tumorigenesis in mice [35], we asked whether RTA 405-mediated activation of NRF2 could increase survival of cells with mutant *Kras*. To investigate this, we first used MEFs derived from LSL-Kras^G12D/+^ mice [50]. Treatment with adenoviral Cre recombinase resulted in complete excision of the STOP cassette (Fig 8A), which allowed translation of the Kras^G12D^ oncogenic protein (Fig 8B). Compared to mock infected LSL-Kras^G12D/+^ cells, viral-cre infected LSL-*Kras* ^G12D/+^ cells tended to have slightly higher *Nfe2l2* mRNA levels, although the difference was not statistically significant (Fig 8C). However, in our hands, they did not exhibit higher *Nqo1*, *Gclc*, *Gclm*, or *Hmox1* mRNA levels. RTA 405 treatment increased expression of NRF2 target genes similarly in mock infected LSL-Kras^G12D/+^ and viral-Cre infected LSL-Kras^G12D/+^ cells, indicating that RTA 405 activates NRF2 equivalently in both cell lines (Fig 8D and S12 Fig). When we assessed viability, we found that RTA 405 inhibited growth equivalently in mock infected LSL-Kras^G12D/+^ and viral-Cre infected LSL-Kras^G12D/+^ MEFs (Fig 8E). We next asked whether the presence of a mutant *KRAS* allele is associated with NRF2 activation in human tumor cell lines. We found that *KRAS* was mutated in a similar percentage of cell lines in the groups with low (50%, *n* = 8) or high (43%, *n* = 7) basal NRF2 activity (S5 Table). In addition, cell lines with mutant *KRAS* exhibited no significant difference in basal levels of *NQO1* mRNA, total glutathione, or ROS when compared to those with wild-type *KRAS* (S13 Fig). Cell lines with wild-type or mutant *KRAS* alleles were equivalently sensitive to RTA 405-mediated growth inhibition and caspase activation (Fig 8F and 8G). Furthermore, activation of NRF2 by a low dose of RTA 405 did not differentially affect survival of cell lines with wild-type or mutant *KRAS* alleles (Fig 8H). We observed similar results in a subset of cell lines treated with bardoxolone methyl (S10 Fig). Taken together, these results indicate that activation of NRF2 by RTA 405 does not increase survival of cells with mutations in *KRAS*.

### RTA 405 treatment does not decrease sensitivity of tumor lines to doxorubicin or cisplatin

**Fig 9:**
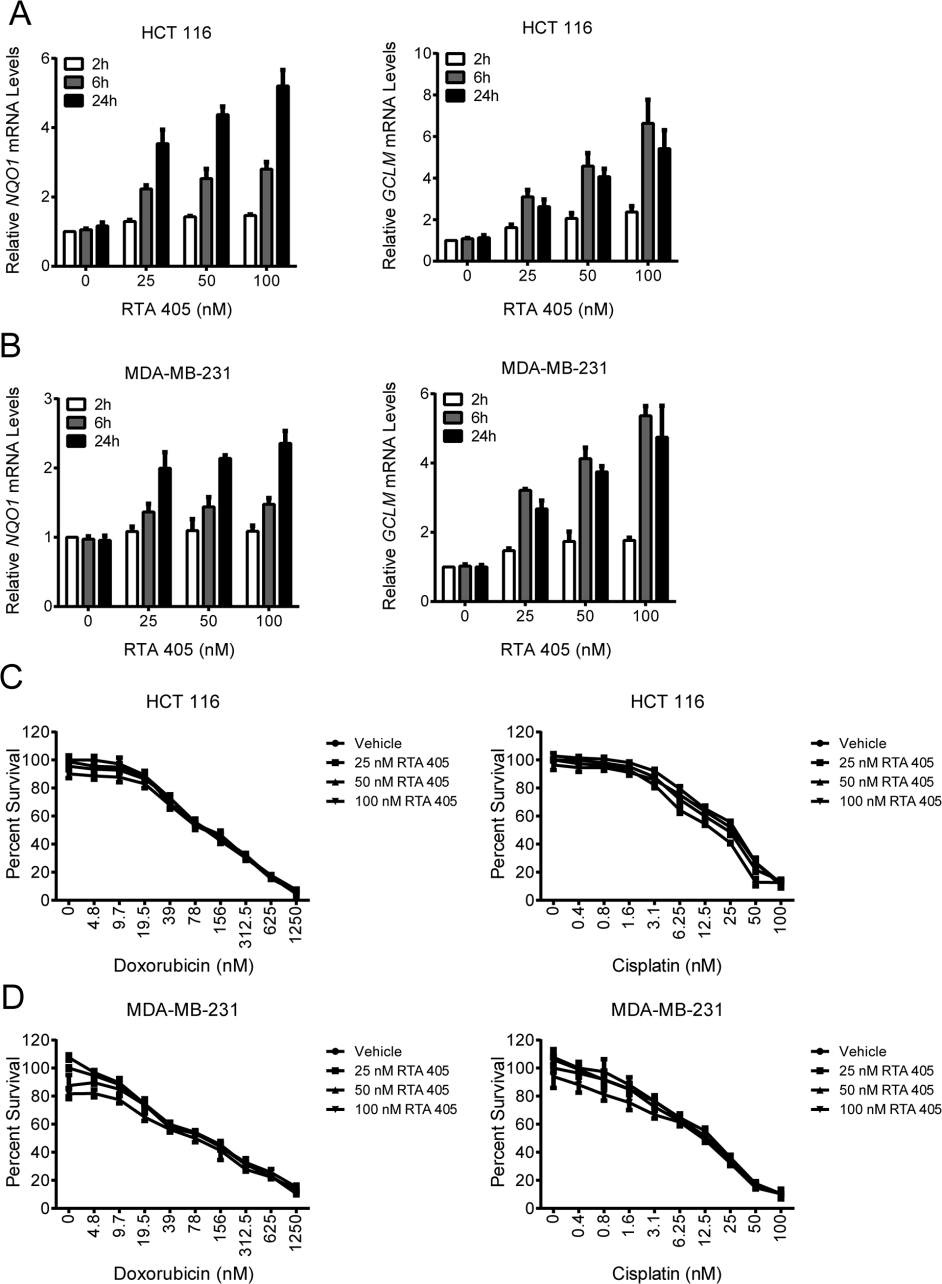
Effect of RTA 405 on doxorubicin- and cisplatin-mediated growth inhibition. **A**-**B**. Effect of RTA 405 treatment on *NQO1* (left panel) and *GCLM* (right panel) mRNA levels in HCT 116 (A) and MDA-MB-231 (B) cells. Cells were treated with the indicated concentrations of RTA 405 for 2, 6, or 24 hours and mRNA levels were assessed by qPCR. Data points are the mean of three independent experiments. Error bars are SD. **C-D.** Effect of RTA 405 treatment on the growth inhibitory activity of doxorubicin (left panel) or cisplatin (right panel) in HCT 116 (C) and MDA-MB-231 (D) cells. Cells were treated with the indicated concentrations of RTA 405 for 24 hours and then treated with doxorubicin or cisplatin for an additional 72 hours. Cell viability was determined using the SRB assay. *Data points*, mean percent survival of triplicates; *error bars*, SD. Data is representative of three individual experiments.

KEAP1 inactivation and NRF2 activation are associated with increased resistance to chemotherapeutics in human tumors [36]. To assess whether activation of NRF2 by RTA 405 reduces the sensitivity of tumor cells to other therapeutic agents we first identified an appropriate duration of RTA 405 pre-treatment. Treatment with 25, 50, or 100 nM RTA 405 for 2, 6, or 24 hours increased *NQO1* and *GCLM* mRNA levels in a time- and dose-dependent manner in HCT 116 (Fig 9A) and MDA-MB-231 (Fig 9B) cells. Despite robust induction of NRF2 target genes, pre-treatment with RTA 405 for 24 hours did not reduce the sensitivity of either cell line to growth inhibition by doxorubicin or cisplatin (Fig 9C and 9D). We observed similar results when cells were pre-treated with RTA 405 for two or six hours (S14 Fig).

## Discussion

**Fig 10:**
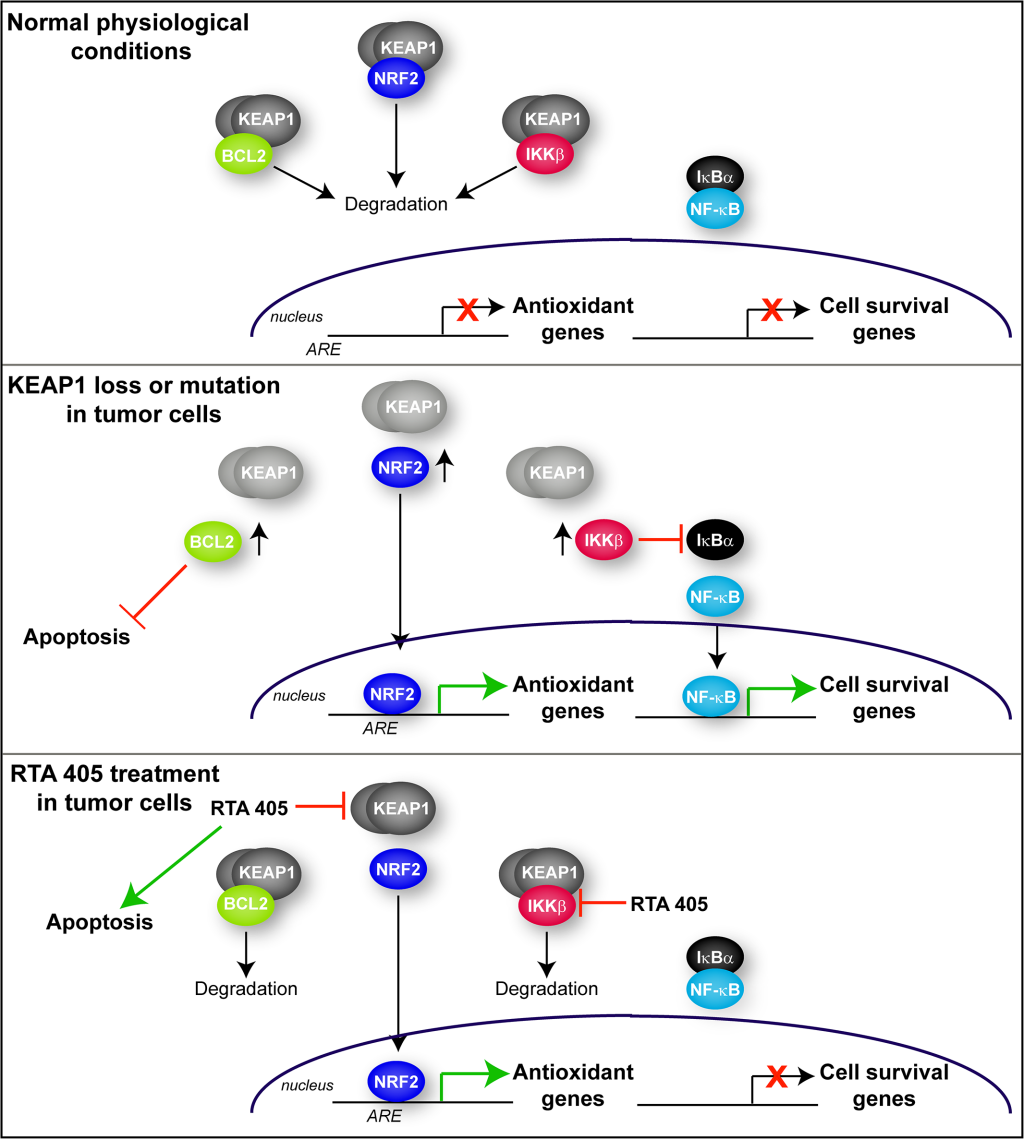
Schematic diagram of the different consequences of genetic loss or mutation of KEAP1 and pharmacological inhibition of KEAP1 by RTA 405. ***Upper panel***. Under normal physiological conditions, KEAP1 promotes the degradation of its target proteins: NRF2, BCL2, and IKKβ. NRF2 target antioxidant genes and NF-κB target cell survival genes are not expressed. ***Middle panel***. When KEAP1 is mutated or KEAP1 levels are reduced, it is no longer able to promote degradation of its target proteins. Therefore NRF2, IKKβ, and BCL2 levels are elevated. As a result, NRF2 accumulates, translocates to the nucleus and increases expression of antioxidant genes. IKKβ levels also accumulate and phosphorylate IκBα, resulting in its degradation. When IκBα is degraded, NF-κB is able to translocate to the nucleus and increase expression of cell survival genes. Elevated BCL2 levels inhibit apoptosis. ***Lower panel***. RTA 405 binds to KEAP1 and blocks its ability to promote NRF2 degradation. NRF2 then translocates to the nucleus where it is transcriptionally active. However, RTA 405 does not inhibit the ability of KEAP1 to promote BCL2 or IKKβ degradation; therefore, the levels of these proteins are not elevated. Furthermore, RTA 405 also directly inhibits the activity of IKKβ, further reducing downstream NF-κB activity and inhibiting NF-κB target gene expression. RTA 405 also increases apoptosis independently of KEAP1/NRF2.

The results from this study demonstrate that treatment with an AIM does not have the same effect as KEAP1 loss or mutation on cancer cell growth and survival. There are several mechanistic differences between these two modes of KEAP1 inhibition that could explain these disparate effects (summarized in Fig 10). The first is the differential effect of RTA 405 on KEAP1-mediated degradation of its target proteins. We found that basal levels of other cancer-related KEAP1 targets, IKKβ and BCL2, were elevated in *Keap1* ^-/-^ MEFs (Fig 3) and in human tumor lines with high basal NRF2 activity (Fig 2). Moreover, in human tumor biopsies, IKKβ protein levels are inversely correlated with KEAP1/CUL3 levels [59;61]. In contrast, RTA 405 increased the levels of NRF2, but not IKKβ or BCL2, in human tumor cell lines (Fig 5). KEAP1-NRF2 binding has recently been shown to involve different amino acids than those involved in the KEAP1-IKKβ interaction [66]. Furthermore, several KEAP1 mutations that were identified in lung cancer specimens differentially affect binding of NRF2 and IKKβ to KEAP1 [57]. These data support a model where AIM binding to KEAP1 induces a conformational change that **blocks** the ability of KEAP1 to promote NRF2 ubiquitination, but **preserves** the ability of KEAP1 to target other proteins, such as IKKβ and BCL2, for ubiquitination. It is interesting to note that *tert*-Butylhydroquinone (tBHQ) increases both NRF2 and BCL2 levels in the Hepa-1 cell line [60], suggesting that distinct classes of KEAP1 ligands may have different effects on KEAP1-interacting proteins.

A second difference between AIMs treatment and and KEAP1 loss or mutation is that the AIMs are also able to directly suppress NF-κB activity by binding to cysteine residue 179 of IKKβ and inhibiting its kinase activity [28;29]. Consistent with this, RTA 405 has previously been shown to inhibit cancer cell growth and induce apoptosis at concentrations that inhibit NF-κB [46;47]. We and others have shown that loss or mutation of KEAP1 increases the levels of NRF2, IKKβ, and downstream NF-κB activity [59]. In contrast, RTA 405 binds to KEAP1 and increases the levels of NRF2, but does not increase IKKβ levels or NF-κB activity. Moreover, RTA 405 also directly inhibits IKKβ and decreases NF-κB activity, as evidenced by reduced Ccnd1 levels in *Keap1* ^*-/-*^ MEFs The decrease in NF-κB activity may suppress tumor cell survival and promote apoptosis (Fig 10).

A third difference between AIM treatment and KEAP1 loss or mutation could be attributed to additional KEAP1-independent effects of AIMs. In this study, we found that RTA 405-mediated inhibition of tumor cell growth (IC_50_ and GI_50_ values) did not correlate with basal NRF2 activity or with the ability of RTA 405 to increase NRF2 activity (Fig 7), consistent with many studies that have shown that RTA 405 modulates the activity of other proteins that directly influence the growth and survival of tumor cells. Indeed several targets and pathways have been reported to contribute to the direct anticancer activity of the AIMs, including IKKβ, STAT3, JNK, CDKN1A (p21), and cyclin D1 [1;67].

A final and important difference between RTA 405 treatment and loss of KEAP1 function is that the latter results in a more robust and prolonged activation of NRF2. RTA 405 treatment increased NRF2 target gene expression to only 27–40% of that in *Keap1* ^-/-^ MEFs (Fig 5A and 5B). Similar results were observed when *Nqo1* mRNA levels were compared in mouse liver tissue from Keap1-KD mice or wild type mice treated with another AIM, CDDO-Im [68]. Compared to the effects of KEAP1 loss, activation of NRF2 target gene expression by AIMs is less robust and may be shorter in duration due to the reversible nature of AIM binding to thiols such as the sensor cysteine (C151) on Keap1 [69]. Alternatively, pharmacological NRF2 activators may engage an auto-regulatory KEAP1-NRF2 feedback loop [70], which would be dysfunctional in the context of KEAP1 deletion or mutation. Therefore, high levels of sustained NRF2 activity, which would occur following KEAP1 loss or mutation but not following pharmacological inhibition of KEAP1, may be required to provide a growth or survival advantage to some cancer cells.

Activated oncogenic proteins such as KRAS^G12D^ have been shown to increase expression of *Nfe2l2* [35;71]. In the present study, expression of KRAS^G12D^ resulted in a small non-statistically significant increase in the mRNA levels of *Nfe2l2*, but not of any NRF2 target genes evaluated (Fig 8C). Increased expression of *Nfe2l2* has previously been proposed to facilitate KRAS-induced proliferation; yet, in the same study, abrogation of the KRAS^G12D^-mediated increase of *Nfe2l2* expression by the MEK inhibitor AZD6244 did not inhibit the KRAS^G12D^-mediated increase in proliferation [35]. Consistent with this, we found that RTA 405 treatment did not increase proliferation of KRAS^G12D^ MEFs (Fig 8E). Similar findings have also been reported in vivo: chronic administration of a natural NRF2 activator, sulforaphane, did not enhance tumorigenesis in the KRAS^G12D^ mouse lung cancer model, despite increasing the levels of NRF2 target genes in lung tissue [72]. Similarly, treatment with bardoxolone methyl increased survival in the *LSL-Kras* ^*G12D/+*^;*LSL-Trp53* ^*R172H/+*^;*Pdx-1-Cre* (KPC) mouse model of pancreatic cancer [46].

In this study we found that RTA 405 did not reduce the sensitivity of tumor cells to doxorubicin- or cisplatin-mediated growth inhibition, despite increasing NRF2 activity. This is consistent with a recent study, which demonstrated that the efficacy of cisplatin and paclitaxel in a mouse lung cancer model was not compromised by bardoxolone methyl [73]. At higher concentrations, AIMs have been shown to enhance the cytotoxic activity of chemotherapeutics [15;27]. Such a dual-acting compound, having NRF2-activating and anticancer properties, could provide an advantage in combination with other standard therapeutics by protecting normal cells from drug-associated toxicities without sacrificing anticancer activity. Furthermore, activation of NRF2 in myeloid-derived suppressor cells reverses tumor-mediated immune suppression [19;20]; therefore, the use of an NRF2 activator in combination with targeted cancer immunotherapies may also be a promising approach to cancer treatment.

We selected the AIM RTA 405 for use in this study as a stringent test of a well-characterized NRF2-activating compound that has demonstrated efficacy in several models of inflammatory disease [41–44]. At higher concentrations, RTA 405 has NRF2-independent anticancer activity and is able to suppress oncogenic signaling pathways, including NF-κB and STAT3 [46]. Moreover, RTA 405 has been shown to reduce the number, size, and grade of tumors in a mouse model of lung cancer [11;47]. The potent anticancer activity of RTA 405 in vivo is likely due its effects on multiple cell types within the tumor microenvironment. In addition to directly inhibiting tumor cell growth, AIMs potently modulate the activity of myeloid-derived suppressor cells and tumor-suppressed T cells [19;20], dendritic cells [18], bone marrow stromal cells [15], macrophages [21], and vascular endothelia [17]. Activation of NRF2 in myeloid-derived suppressor cells prevents metastasis and reverses tumor-mediated immune suppression [19;20;74]; therefore, a compound that activates NRF2 in the tumor microenvironment and directly inhibits tumor cell growth is a promising therapeutic approach. In this regard, bardoxolone methyl was well-tolerated in a phase 1 clinical trial in patients with solid tumors and lymphomas [3]. In this trial, bardoxolone methyl increased the expression of *NQO1* in peripheral blood mononuclear cells and decreased NF-κB and cyclin D1 levels in tumor biopsies. One patient with mantle cell lymphoma had a complete response and a patient with anaplastic thyroid carcinoma had a partial response of 18 months in duration. Based on these promising preliminary findings, the safety and tolerability of a related compound, RTA 408 [2], is currently being evaluated in patients with metastatic non-small cell lung cancer or melanoma [75].

## Supporting Information

S1 Fig Basal levels of NRF2, KEAP1, and NQO1 proteins in a panel of human tumor cell lines (uncropped blots). (TIF)

S2 Fig Basal levels of *NQO1* mRNA, total glutathione, and reactive oxygen species in a panel of human tumor cell lines. (TIF)

S3 Fig Protein levels of other KEAP1-interacting proteins. (TIF)

S4 Fig Characterization of wild-type and *Keap1* ^-/-^ murine embryonic fibroblasts. (TIF)

S5 Fig Basal mRNA levels of *Ccl5*, *Bcl2l1*, *Birc3*, *Ccl2*, and *Il1b* in wild-type and *Keap1* ^-/-^ MEFs measured by qPCR. (TIF)

S6 Fig Growth rates of human tumor cell lines with different levels of basal NRF2 activity. (TIF)

S7 Fig Validation of NRF2 knockdown by siRNA. (TIF)

S8 Fig *NQO1* and *GCLM* mRNA levels following treatment with RTA 405. (TIF)

S9 Fig Effect of RTA 405 on other KEAP1 target proteins. (TIF)

S10 Fig Basal NRF2 activity and KRAS genotype does not affect bardoxolone methyl-mediated inhibition of cell growth and viability. (TIF)

S11 Fig Effect of RTA 405 on markers of apoptosis and cell cycle control. (TIF)

S12 Fig Effect of RTA 405 treatment on *Hmox1*, *Gclc*, and *Nrf2* mRNA levels in LSL-*Kras* ^G12D/+^ murine embryonic fibroblasts. (TIF)

S13 Fig Assessment of basal NRF2 activity in a panel of human tumor cell lines with wild-type or mutant *KRAS*. (TIF)

S14 Fig Effect of RTA 405 treatment on the growth inhibitory activity of doxorubicin or cisplatin in HCT-116 and MDA-MB-231 cells. (TIF)

S1 Protocols Additional Materials and Methods. (DOCX)

S1 Table PCR primer information. (DOCX)

S2 Table Antibody information. (DOCX)

S3 Table Effect of AIMs on Viability, Growth, and Apoptosis in Human Tumor Cell Lines. (DOCX)

S4 Table Effect of RTA 405 on Markers of Apoptosis and Proliferation in Human Tumor Cell Lines. (DOCX)

S5 Table *KRAS* Status in Human Tumor Cell Lines. (DOCX)
