# Supplementary figures and images for: Cancer Cell Growth Is Differentially Affected by Constitutive Activation of NRF2 by KEAP1 Deletion and Pharmacological Activation of NRF2 by the Synthetic Triterpenoid, RTA 405
Source: PLoS One. 2015 Aug 24;10(8):e0135257. doi: 10.1371/journal.pone.0135257 (PMC4547720; doi:10.1371/journal.pone.0135257)

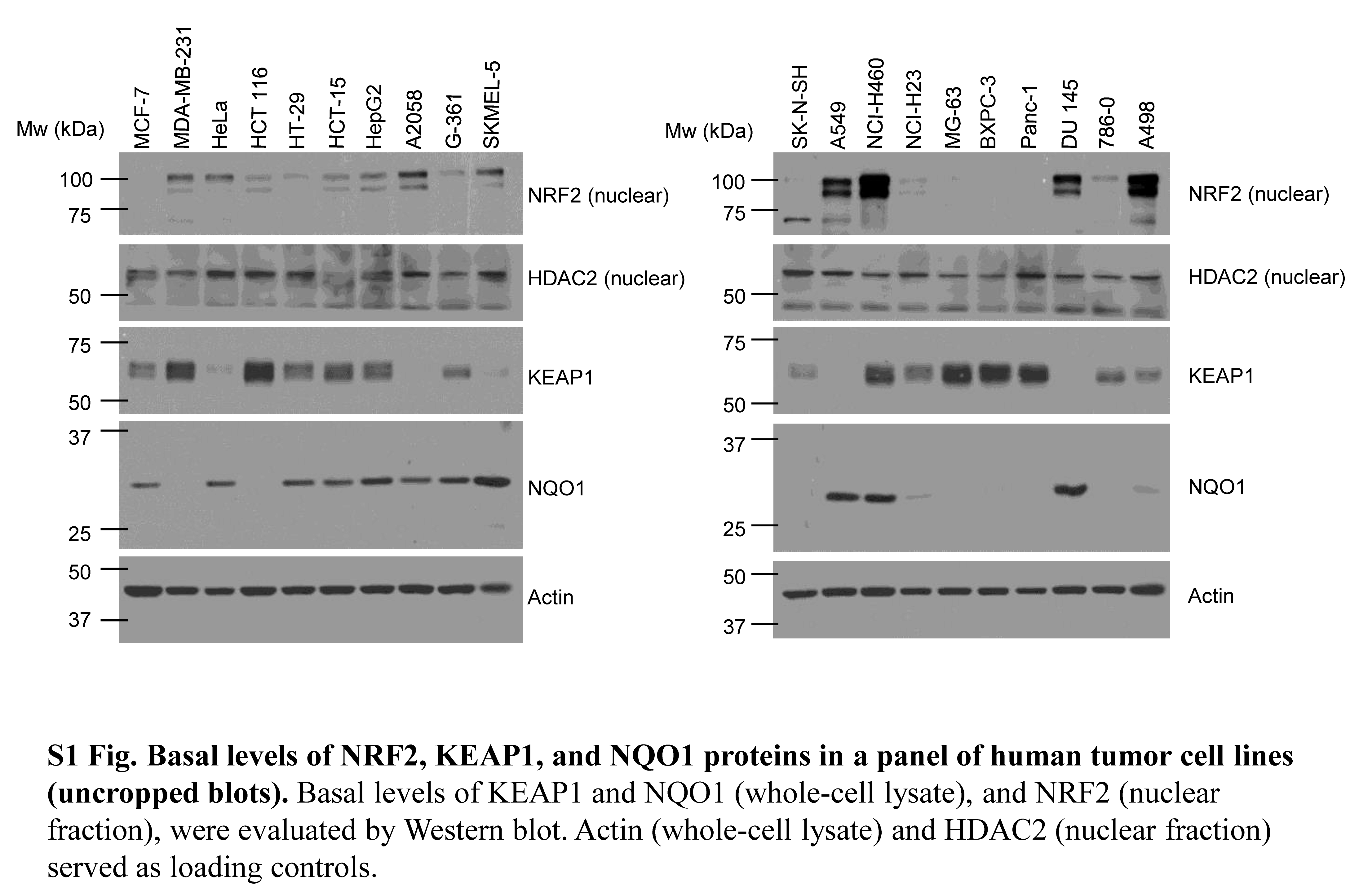

Supplement: S1 Fig — (TIF) [file pone.0135257.s001.tif]

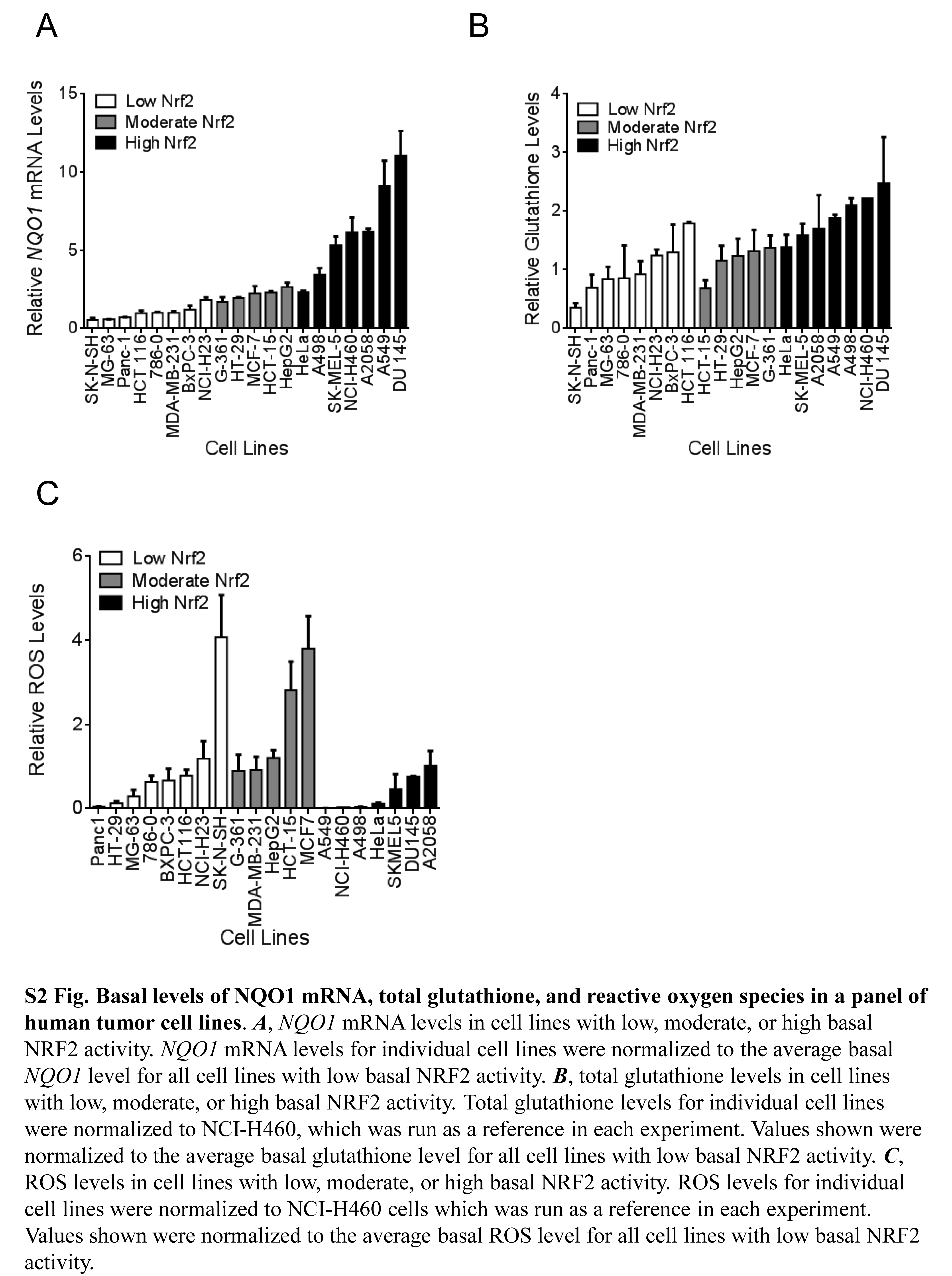

Supplement: S2 Fig — (TIF) [file pone.0135257.s002.tif]

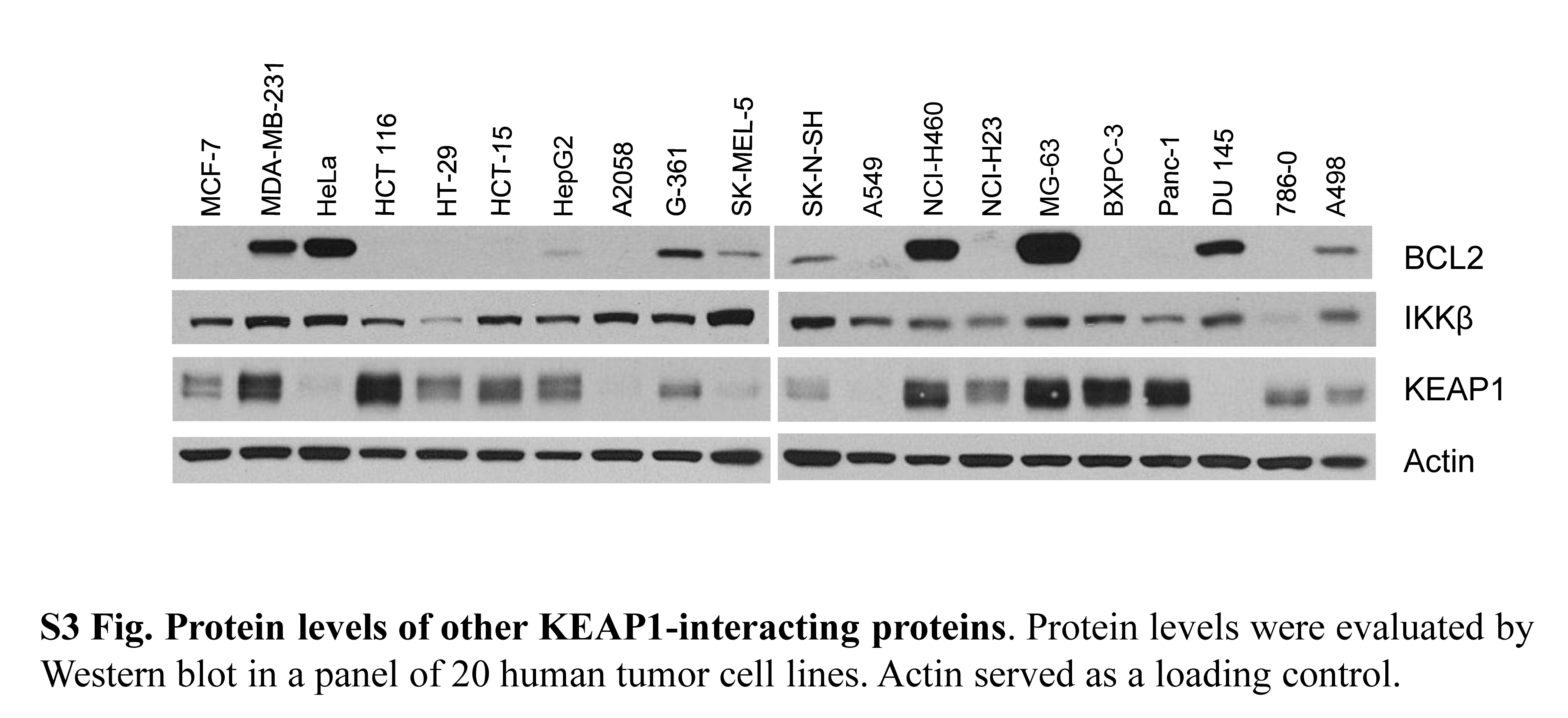

Supplement: S3 Fig — (TIF) [file pone.0135257.s003.tif]

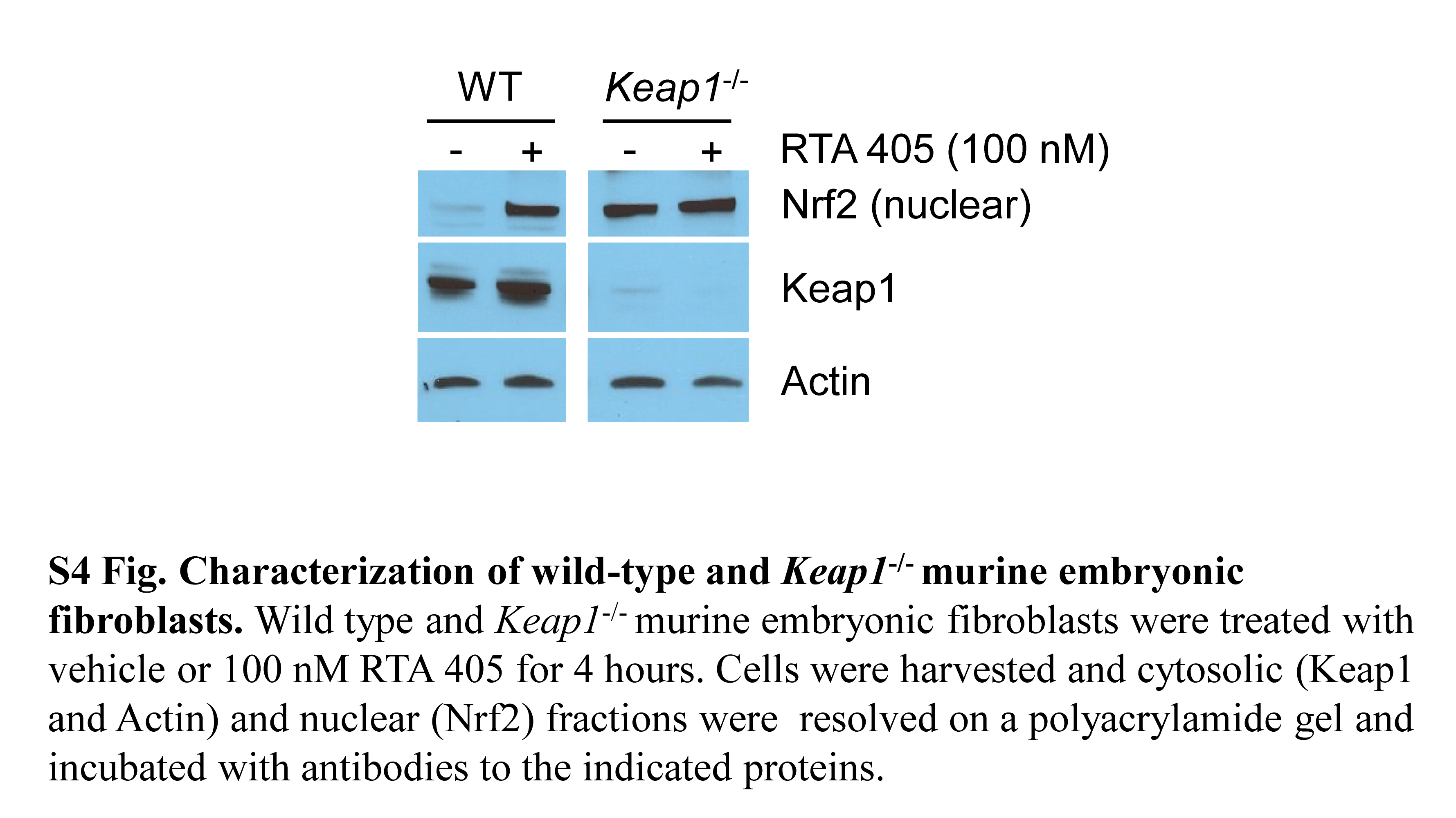

Supplement: S4 Fig — (TIF) [file pone.0135257.s004.tif]

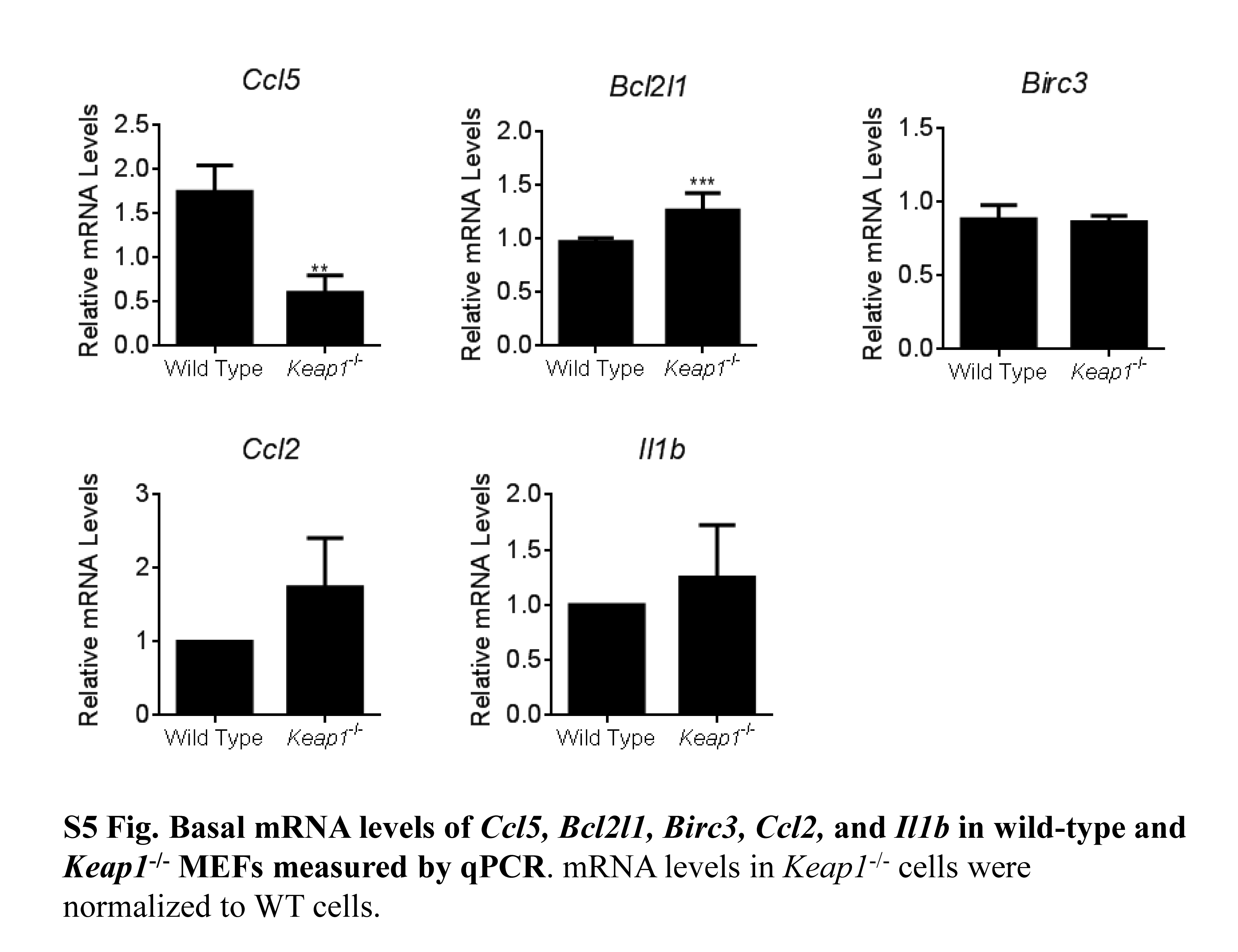

Supplement: S5 Fig — (TIF) [file pone.0135257.s005.tif]

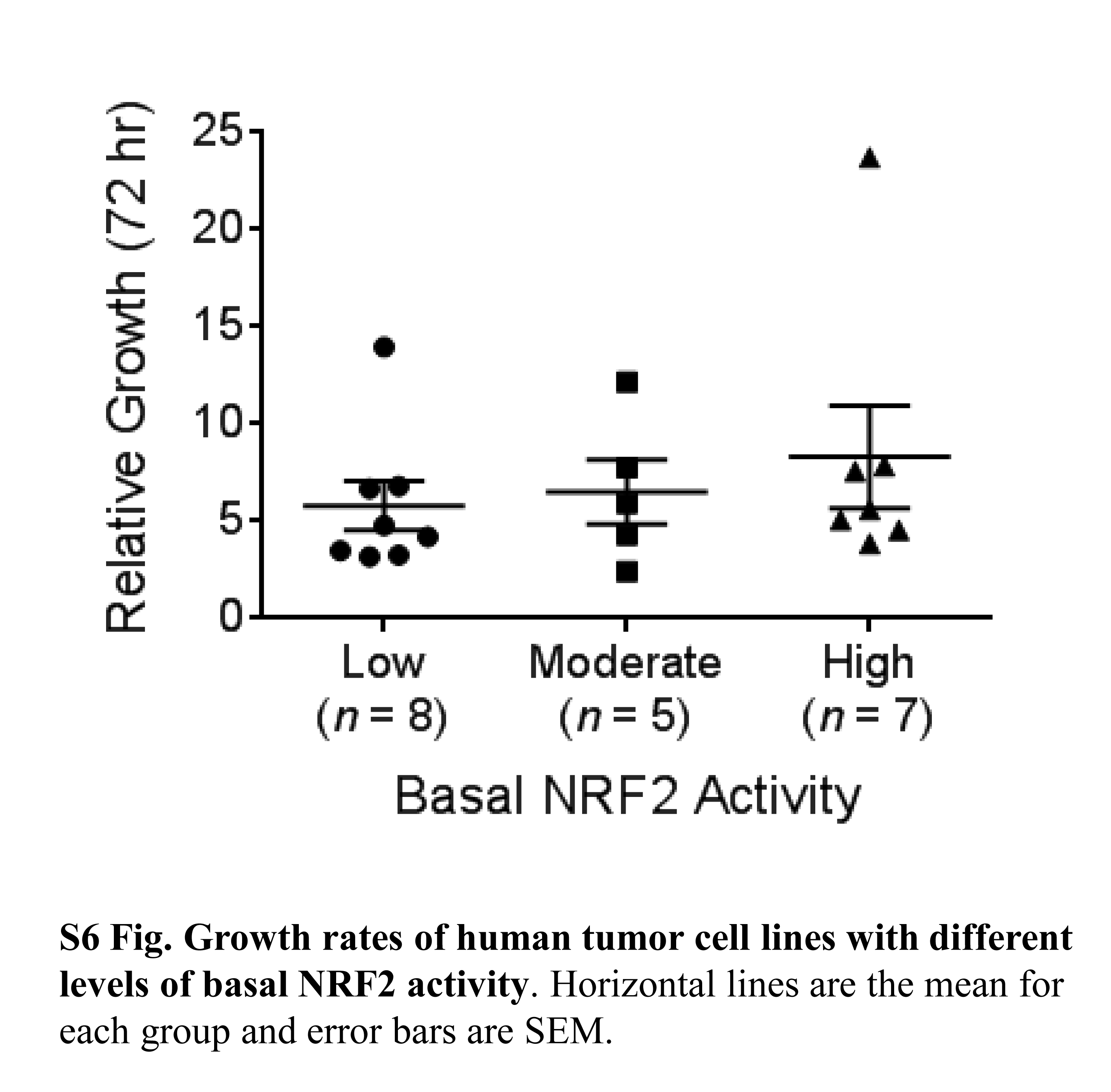

Supplement: S6 Fig — (TIF) [file pone.0135257.s006.tif]

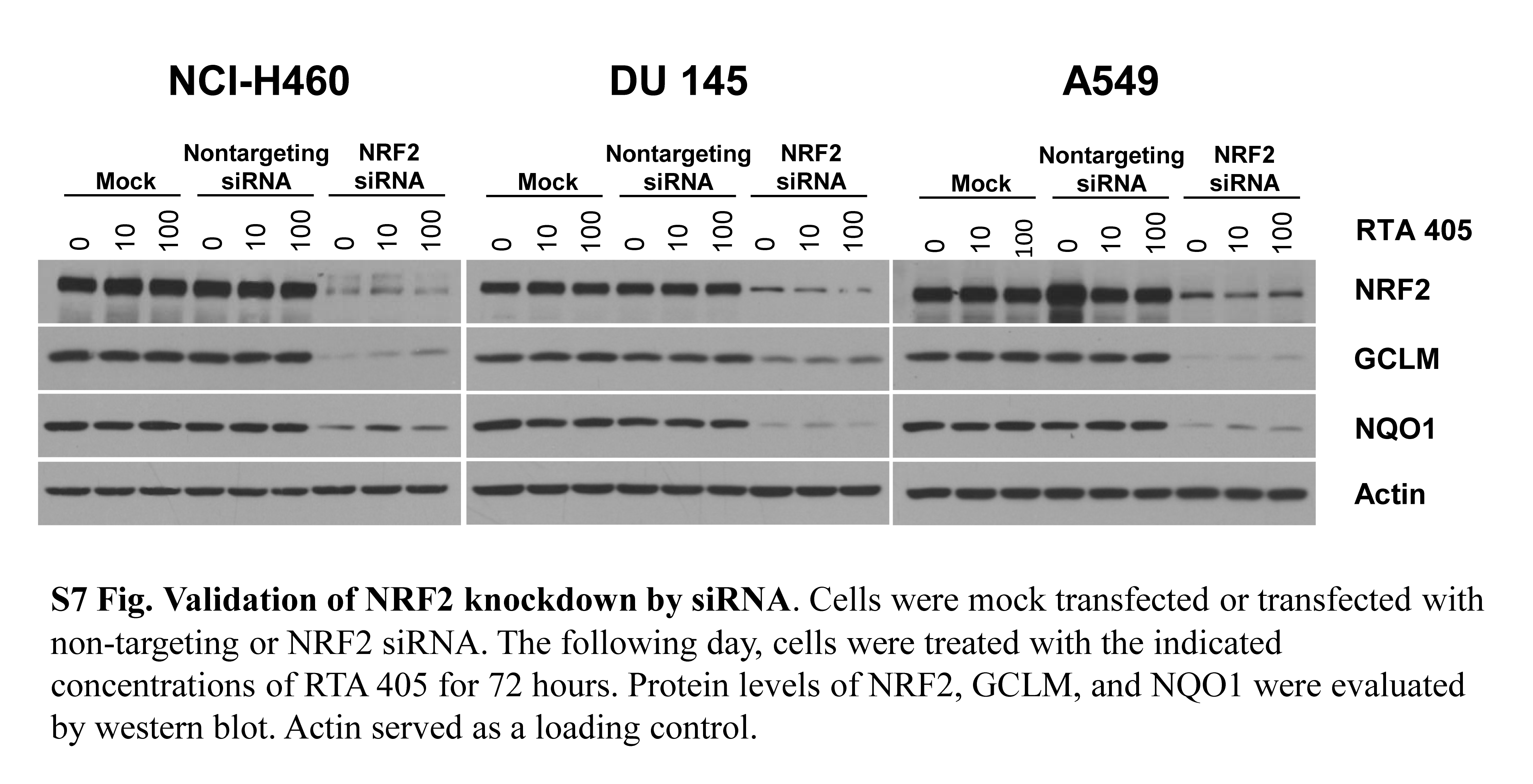

Supplement: S7 Fig — (TIF) [file pone.0135257.s007.tif]

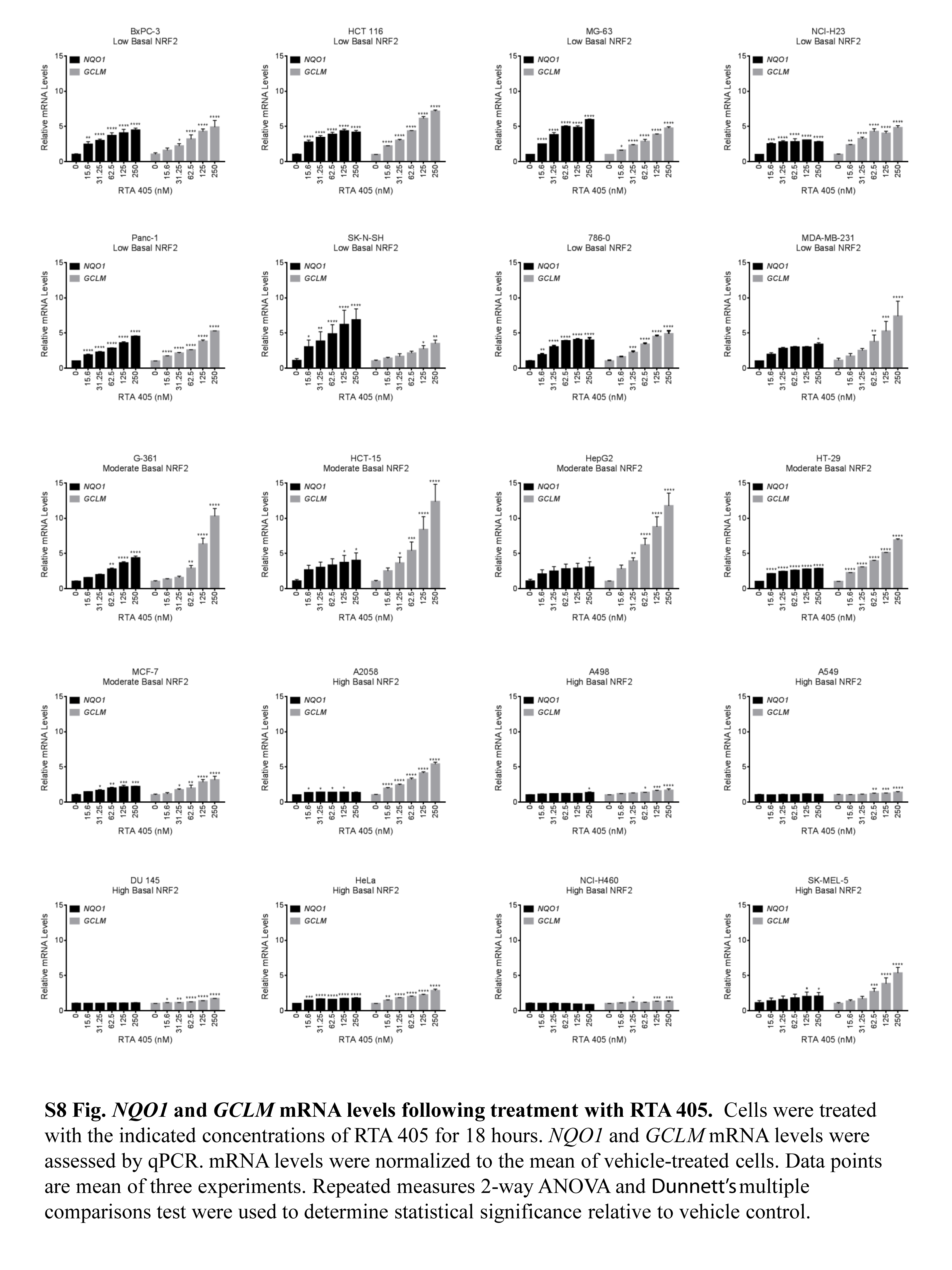

Supplement: S8 Fig — (TIF) [file pone.0135257.s008.tif]

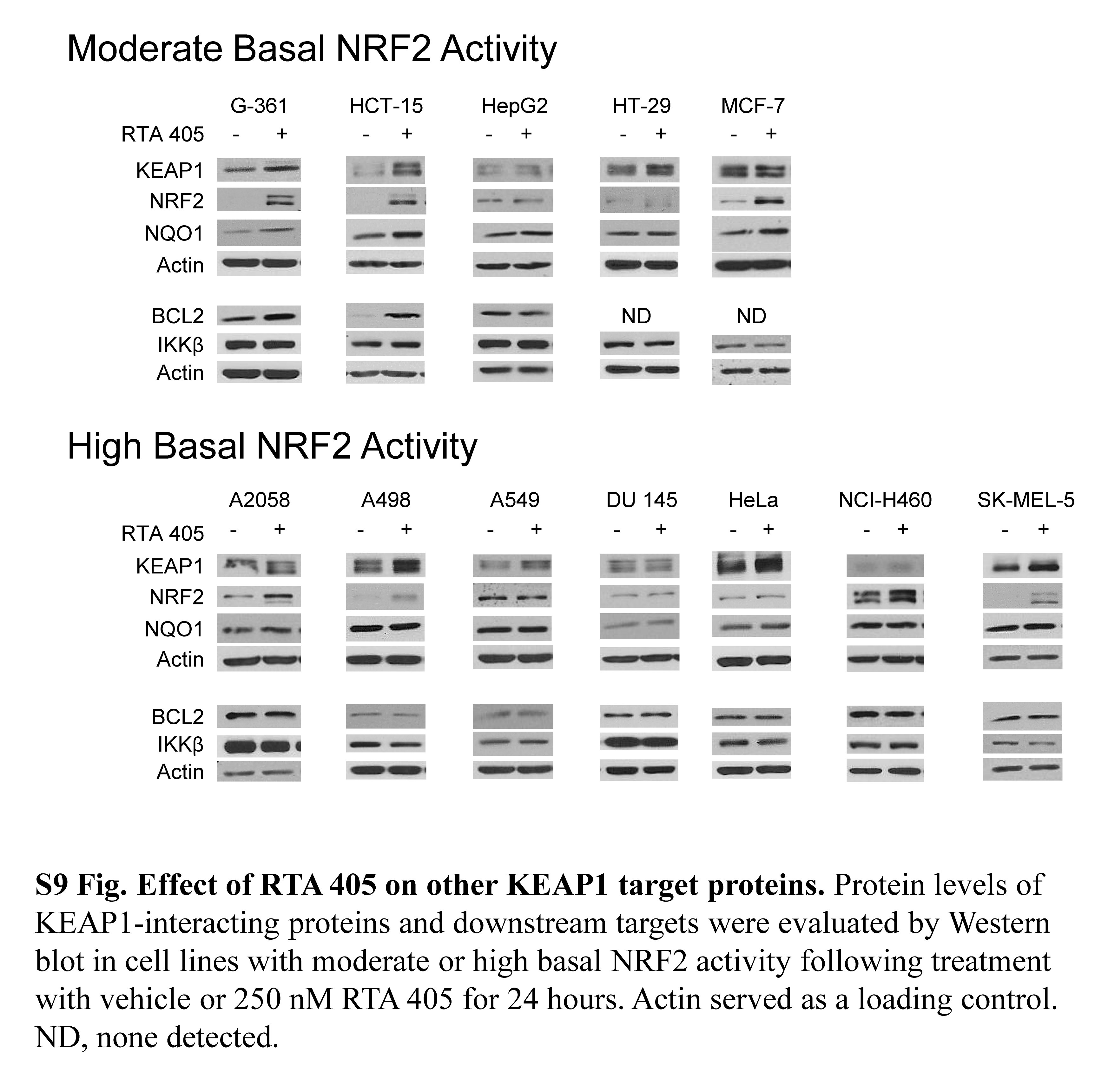

Supplement: S9 Fig — (TIF) [file pone.0135257.s009.tif]

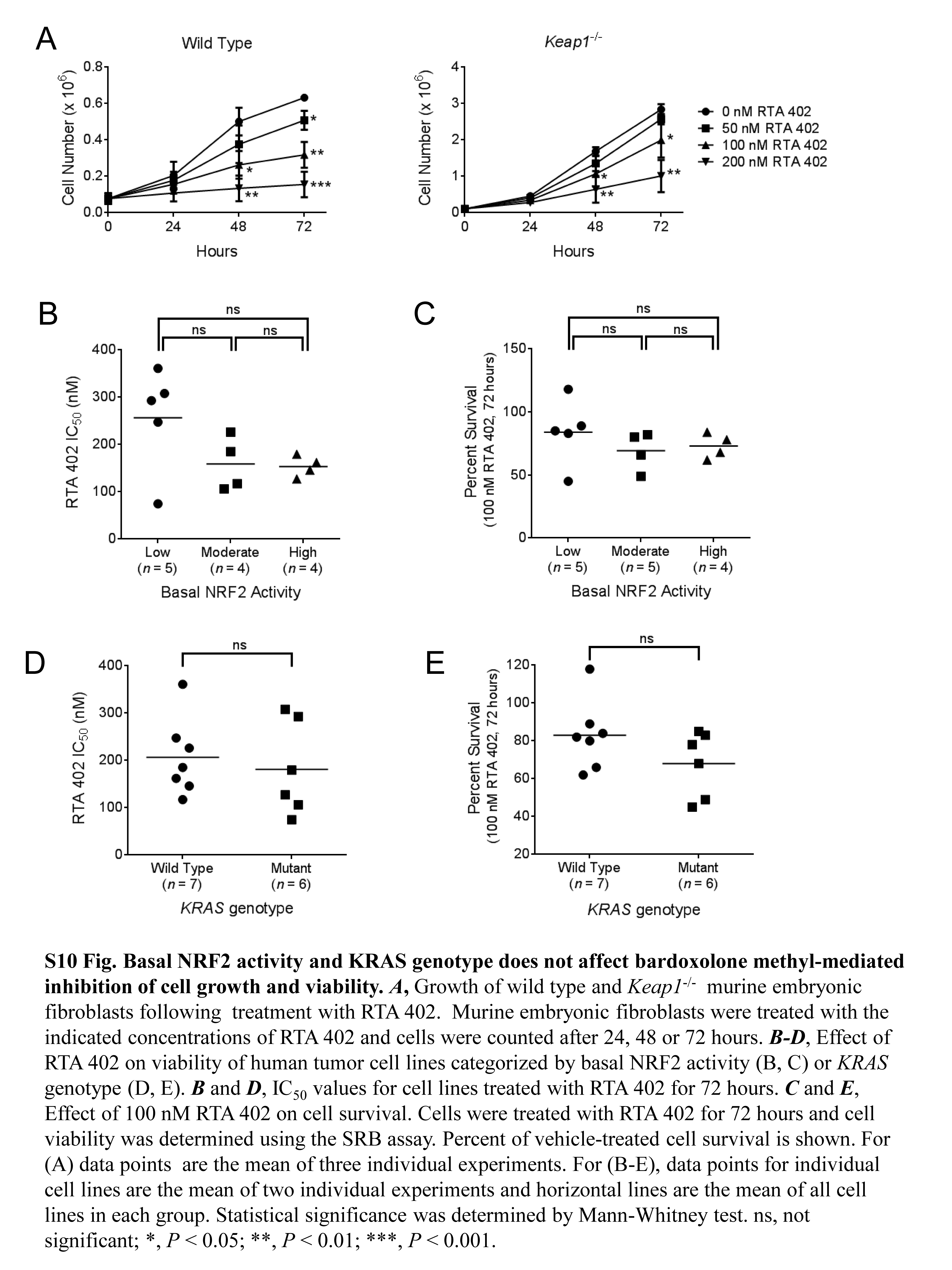

Supplement: S10 Fig — (TIF) [file pone.0135257.s010.tif]

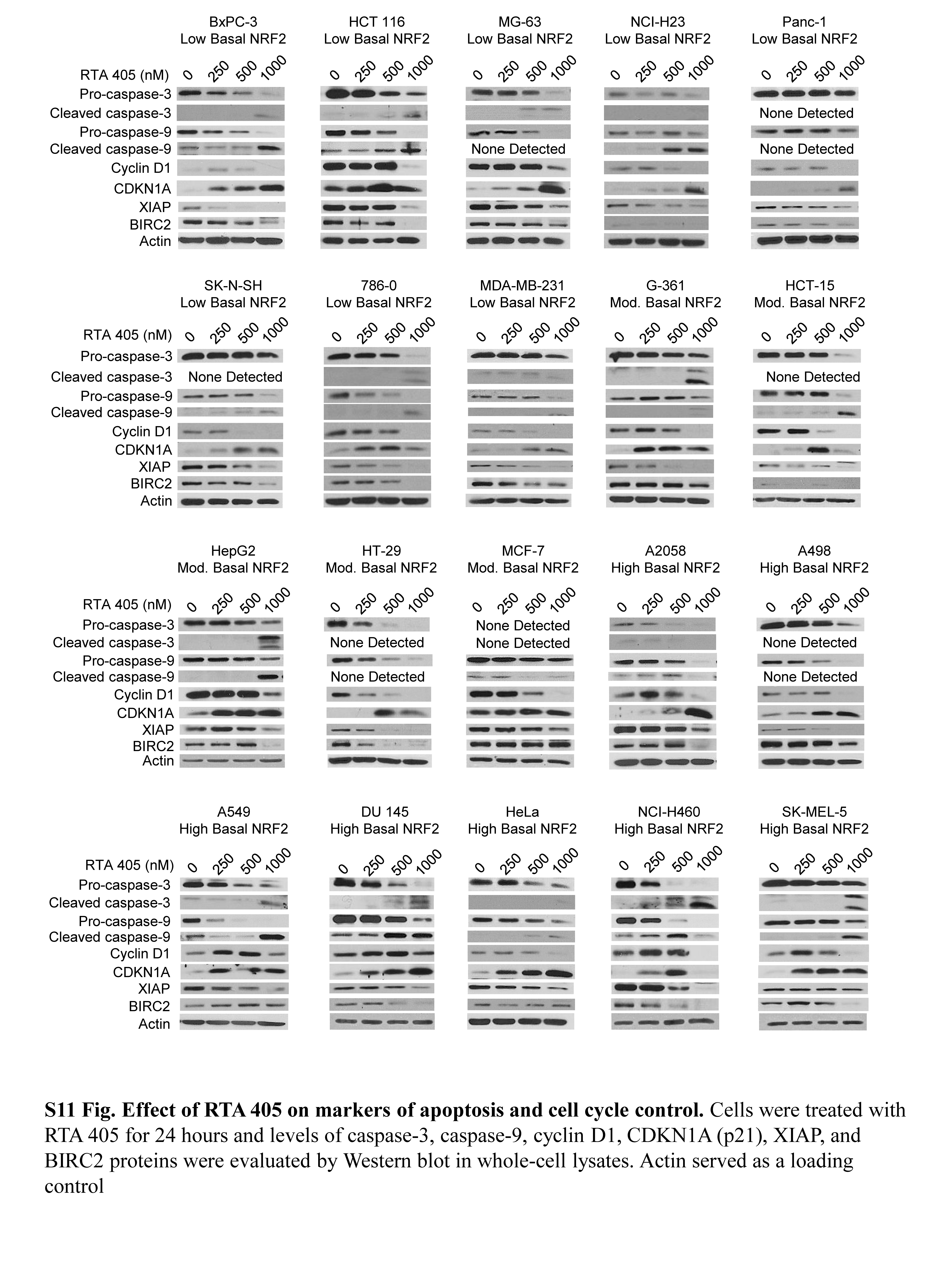

Supplement: S11 Fig — (TIF) [file pone.0135257.s011.tif]

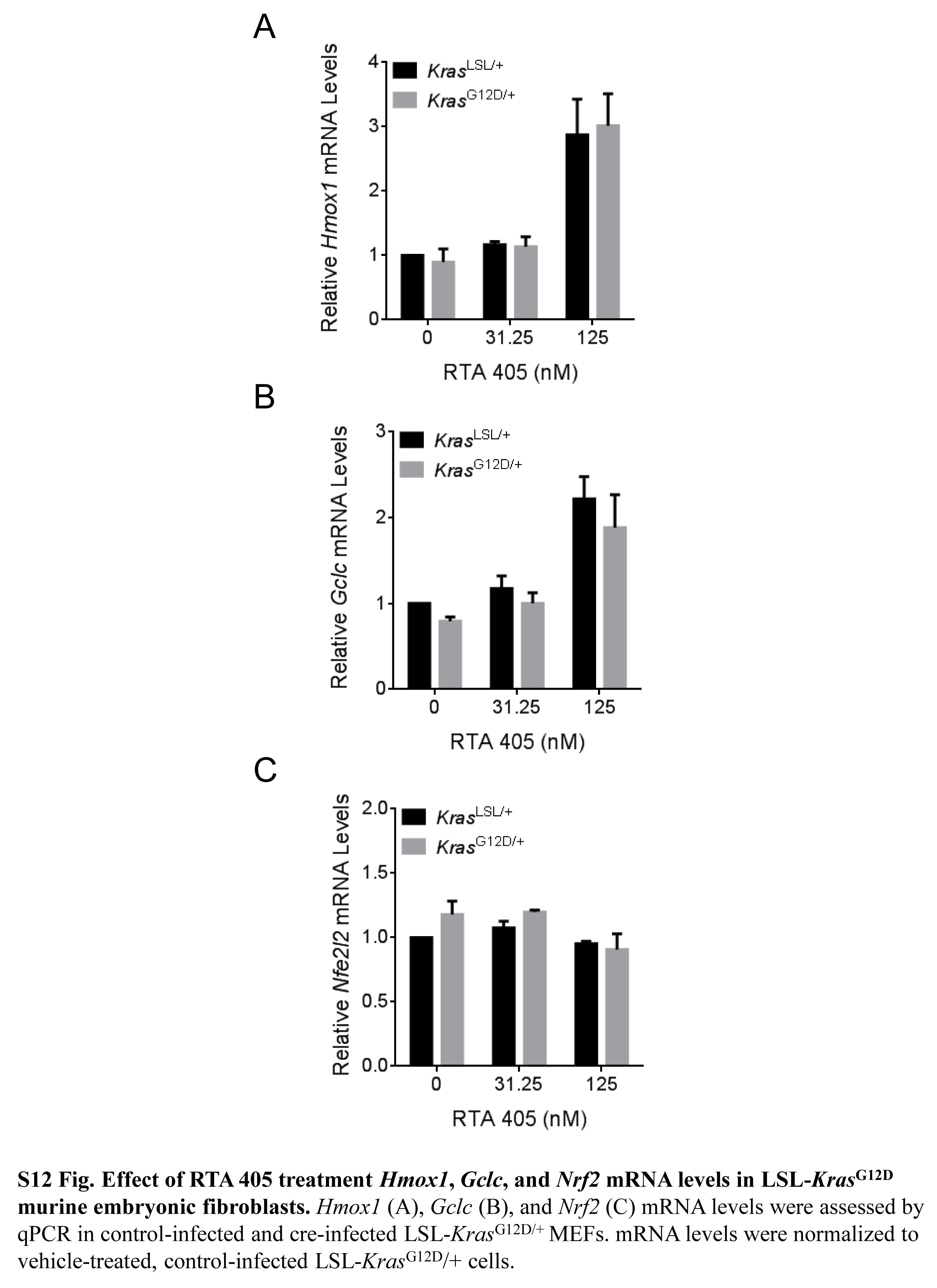

Supplement: S12 Fig — (TIF) [file pone.0135257.s012.tif]

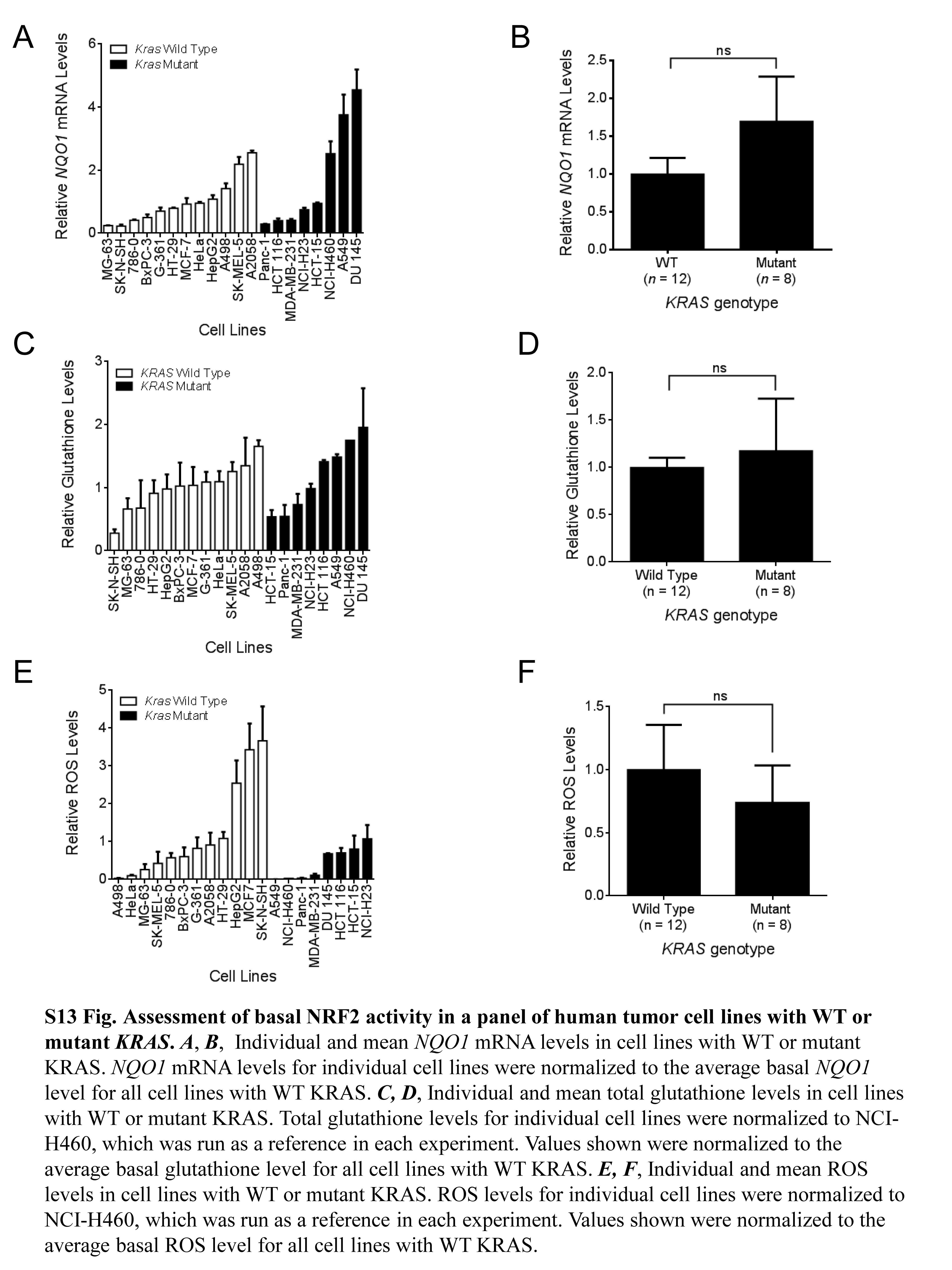

Supplement: S13 Fig — (TIF) [file pone.0135257.s013.tif]

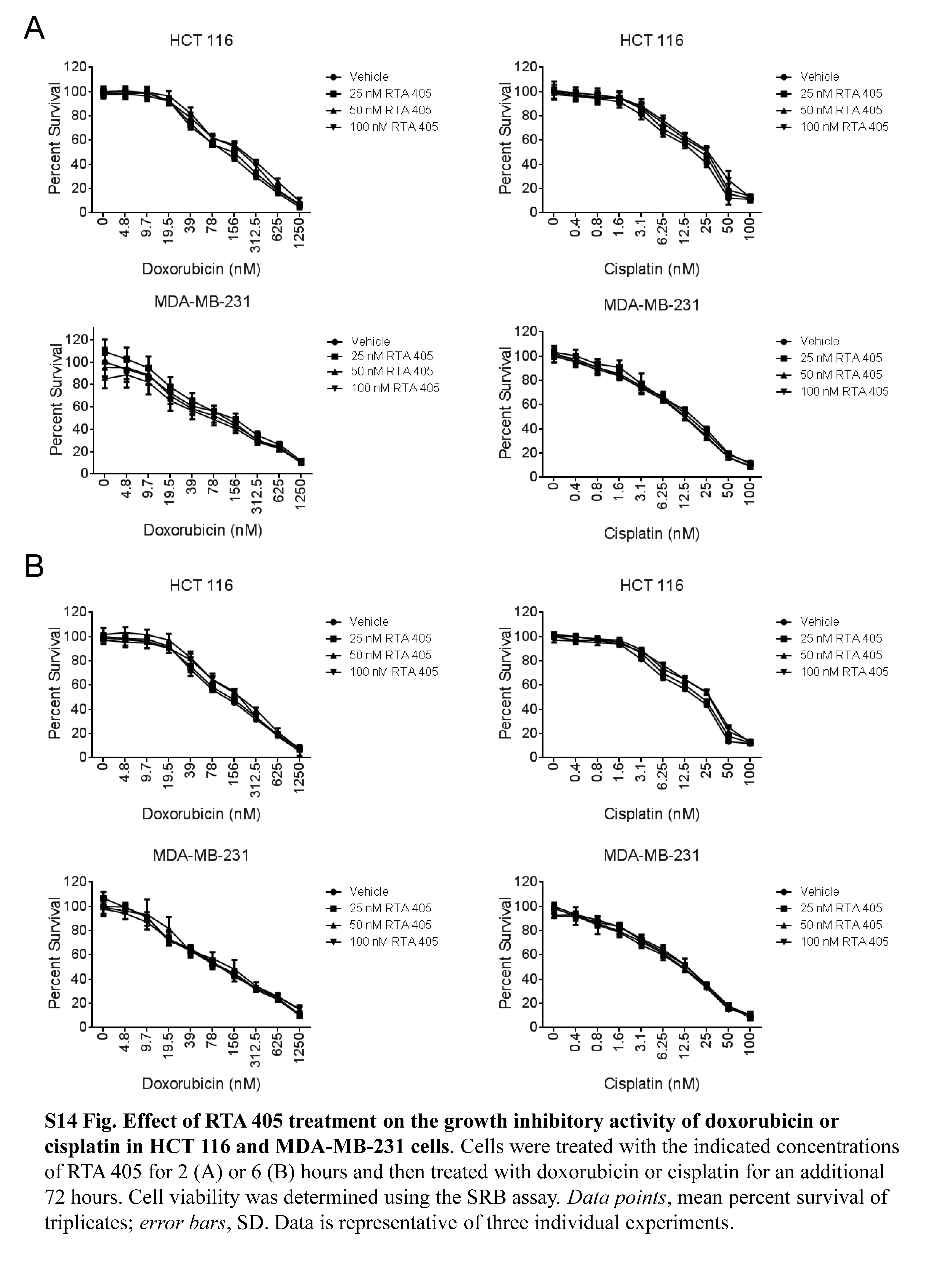

Supplement: S14 Fig — (TIF) [file pone.0135257.s014.tif]
