# Supplementary material for: Cancer Cell Growth Is Differentially Affected by Constitutive Activation of NRF2 by KEAP1 Deletion and Pharmacological Activation of NRF2 by the Synthetic Triterpenoid, RTA 405
Source: PLoS One. 2015 Aug 24;10(8):e0135257. doi: 10.1371/journal.pone.0135257 (PMC4547720; doi:10.1371/journal.pone.0135257)
Supplement: S1 Protocols — (DOCX) [file pone.0135257.s015.docx]

**File S1. Additional Materials and Methods**

**Reverse transcription and PCR.** Reverse transcription was performed with 1 µg of RNA, oligo(dT)_12-18_ primer, and Superscript II reverse transcriptase (Life Technologies). Real-time PCR was performed using iQ™ SYBR^®^ Green Supermix in a CFX96™ Real-Time PCR Detection System (Bio-Rad). PCR amplification of genomic DNA was performed using Vent (New England Biolabs) or Taq (Life Technologies) DNA polymerase, dNTPs, and 0.2 µM of each primer. The PCR cycling conditions were as follows: 94°C (5 min) for one cycle; 94°C (30 sec), 58°C (45 sec), and 72°C (1 min) for 40 cycles; and 72°C (5 min) for one cycle.

**Preparation of cell lysates for western blot.** To prepare cell lysates, cells were scraped into 1 mL media, centrifuged at 2 000 x *g*, and washed with PBS. Pellets were resuspended in lysis buffer containing 20 mM HEPES (pH 7.4), 1.5 mM MgCl_2_, 1 mM DTT, 10 mM KCl, 1 mM EGTA, 1 mM EDTA, 1% Triton X-100, Complete Protease Inhibitor Cocktail (Roche Applied Science), and Phosphatase Inhibitor Cocktail 3 (Sigma-Aldrich).

**Preparation of nuclear extracts for western blot.** Nuclear extracts were prepared as described (34) with minor modifications. Cells were washed twice with ice-cold PBS and resuspended in 300 µL of nuclear extraction Buffer A (10 mM HEPES [pH 7.4], 10 mM KCL, 1.5 mM MgCl_2_, 1 mM DTT, and Complete Protease Inhibitor Cocktail). After 15 minutes on ice, 18.75 µL of 10% NP-40 was added and the samples were vortexed. Nuclei were collected by centrifugation at 3 000 x *g* for 5 minutes at 4°C and washed twice with Buffer A. The nuclear pellet was resuspended in 75 µL of nuclear extraction Buffer B (20 mM HEPES [pH 7.4], 400 mM NaCl, 1.5 mM MgCl_2_, 0.2 mM EDTA, 1 mM DTT, 5% glycerol, and Complete Protease Inhibitor Cocktail) and incubated for 30 minutes at 4°C, followed by centrifugation at 10 000 x *g* for 5 minutes at 4°C.

**ROS levels**. To measure basal ROS levels, 3 x 10^5^ cells/well were plated in 6-well dishes. Twenty-four hours later, cells were loaded with 5 µM CM-H_2_DCFDA (Molecular Probes) in pre-warmed HBSS without phenol red at 37°C in 5% CO_2_ for 2 hours. Cells without CM-H_2_DCFDA were included in each experiment to control for background fluorescence. Following incubation, medium was removed and cells were incubated in phenol-red free medium for 10 minutes at 37°C. Cells were then scraped into medium, centrifuged at 1500 rpm, washed with PBS, and resuspended in 500 µl of PBS. ROS levels were determined by measuring the mean fluorescence intensity of 10 000 events by flow cytometry on an Accuri C6 cytometer using CFlow Plus software (Accuri Cytometers, Ann Arbor, MI USA). To control for variability between experiments, the mean fluorescence intensity of each cell line was normalized to that of NCI-H460 (set to a value of 1).

**Glutathione levels**. To determine total glutathione levels, cells were seeded in 96-well tissue culture plates at 5 x 10^3^ cells/well. The next day, total glutathione levels were measured using the GSH-Glo™ Glutathione Assay (Promega) following the manufacturer’s protocol. Glutathione levels were normalized to cellular protein levels as determined using the SRB assay. To control for variability between experiments, the total glutathione level for each cell line was normalized to that of NCI-H460 (set to a value of 1).
