# Supplementary material for: Cancer Cell Growth Is Differentially Affected by Constitutive Activation of NRF2 by KEAP1 Deletion and Pharmacological Activation of NRF2 by the Synthetic Triterpenoid, RTA 405
Source: PLoS One. 2015 Aug 24;10(8):e0135257. doi: 10.1371/journal.pone.0135257 (PMC4547720; doi:10.1371/journal.pone.0135257)
Supplement: S1 Table — (DOCX) [file pone.0135257.s016.docx]

**S1 Table. PCR primer information**

| **Species** | **Gene Symbol** | **Forward primer** | **Reverse primer** |
| --- | --- | --- | --- |
| Human | *RPS9* | 5’-GATGAGAAGGACCCCACGGCGTCTG-3’ | 5’-GAGACAATCCAGCAGCCCAGGAGGG-3’ |
| Human | *NQO1* | 5’-AAAACACTGCCCTCTTGTGG-3’ | 5’-GTGCCAGTCAGCATCTGGTA-3’ |
| Human | *GCLM* | 5’-GCTGTGGCTACTGCGGTATT-3’ | 5’-ATCTGCCTCAATGACACCAT-3’ |
| Human | *HMOX1* | 5’-TCCGATGGGTCCTTACACTC-3’ | 5’-TAGGCTCCTTCCTCCTTTCC-3’ |
| Human | *GCLC* | 5’-CTTGTAGTCAGGATGGTTTGCG-3’ | 5’-TCCTGGACTGATCCCAATTCTG-3’ |
| Human | *TXNRD1* | 5’-ATTGCCACTGGTGAAAGACC-3’ | 5’-ACCAATTTTGTTGGCCATGT-3’ |
| Mouse | *Rpl19* | 5’-TCAGGCTACAGAAGAGGCTTGC-3’ | 5’-ACAGTCACAGGCTTGCGGATG-3’ |
| Mouse | *Nqo1* | 5’-TCGGGCTAGTCCCAGTTAGA-3’ | 5’-AAAGAGCTGGAGAGCCAACC-3’ |
| Mouse | *Gclm* | 5’-GCCACCAGATTTGACTGCCTTTG-3’ | 5’-TGCTCTTCACGATGACCGAGTACC |
| Mouse | *Bcl2l1* | 5’-GCTGGGACACTTTTGTGGAT-3’ | 5’-TGTCTGGTCACTTCCGACTG-3’ |
| Mouse | *Ccnd1* | 5’-CCAACAACTTCCTCTCCTGCT-3’ | 5’-GACTCCAGAAGGGCTTCAATC-3’ |
| Mouse | *Ptgs2* | 5’-TAGGCTGTTGGAATTTACGC-3’ | 5’-TCATTTCTTGATGCCCGAAT-3’ |
| Mouse | *Mmp9* | 5’-CAGGGAGATGCCCATTTCG-3’ | 5’-GGGCACCATTTGGAGTTTCCA-3’ |
| Mouse | *Ccl5* | 5’-GTGCCCACGTGAAGGAGTAT-3’ | 5’-ATCCCCAGCTGGTTAGGACT-3’ |
| Mouse | *Birc3* | 5’-TTTTGAATGCTGAAGATGAGAGA-3’ | 5’-CTGTTGAAAGAGGGCCATTC-3’ |
| Mouse | *Ccl2* | 5’-ATGCAGTTAATGCCCCACTC-3’ | 5’-TTCCTTATTGGGGTCAGCAC-3’ |
| Mouse | *Vegf* | 5’-TGGAAGAAGAGGCCTGGTAA-3’ | 5’-AAGCCACTCACACACACAGC-3’ |
