# Supplementary material for: Cancer Cell Growth Is Differentially Affected by Constitutive Activation of NRF2 by KEAP1 Deletion and Pharmacological Activation of NRF2 by the Synthetic Triterpenoid, RTA 405
Source: PLoS One. 2015 Aug 24;10(8):e0135257. doi: 10.1371/journal.pone.0135257 (PMC4547720; doi:10.1371/journal.pone.0135257)
Supplement: S2 Table — (DOCX) [file pone.0135257.s017.docx]

**S2 Table. Antibody information**

| **Antibody Name** | **Type** | **Host species** | **Dilution** | **Commercial Supplier/Location** | **Catalog Number** |
| --- | --- | --- | --- | --- | --- |
| Actin, clone C4 | Monoclonal | Mouse | 1:100,000 | Millipore/ Billerica, MA USA | MAB1501 |
| BCL2 (50E3) | Monocolonal | Rabbit | 1:2000 | Cell Signaling Technology/ Danvers, MA USA | 2870 |
| BIRC2 (cIAP-1) (pan) | Polyclonal | Goat | 1:1000 | R&D Systems/ Minneapolis, MN USA | AF8181 |
| Caspase-3 (8G10) | Monoclonal | Rabbit | 1:1000 | Cell Signaling Technology/ Danvers, MA USA | 9665 |
| CDKN1A (p21) (N-20) | Polyclonal | Rabbit | 1:1000 | Santa Cruz Biotechnology/ Dallas, TX USA | sc-469 |
| Cleaved Caspase-3 (Asp175) | Polyclonal | Rabbit | 1:1000 | Cell Signaling Technology/ Danvers, MA USA | 9661 |
| Caspase-9 | Polyclonal | Rabbit | 1:1000 | Cell Signaling Technology/ Danvers, MA USA | 9502 |
| Cyclin D1 (DCS-6) | Monoclonal | Mouse | 1:1000 | Santa Cruz Biotechnology/ Dallas, TX USA | sc-20044 |
| Heme oxygenase-1 (H-105) | Polyclonal | Rabbit | 1:500 | Santa Cruz Biotechnology/ Dallas, TX USA | sc-10789 |
| IκBα (C-21) | Polyclonal | Rabbit | 1:5000 | Santa Cruz Biotechnology/ Dallas, TX USA | sc-371 |
| IKKβ (2C8) | Monoclonal | Rabbit | 1:3000 | Cell Signaling Technology/ Danvers, MA USA | 2370 |
| Keap1 | Polyclonal | Rabbit | 1:1000 | Proteintech Group/ Chicago, IL USA | 10503-2-AP |
| Kras | Monoclonal | Rabbit | 1:250 | Cell Signaling Technology/ Danvers, MA USA | 3965 |
| Kras^G12D^ | Monoclonal | Mouse | 1:200 | New East Biosciences/ King of Prussia, PA USA | 26036 |
| NQO1 (C-19) | Polyclonal | Goat | 1:1000 | Santa Cruz Biotechnology/ Dallas, TX USA | sc-16464 |
| Nrf2 | Monoclonal | Rabbit | 1:1000 | Epitomics/ Burlingame, CA USA | 2178-1 |
| SQSTM1/p62 | Monoclonal | Rabbit | 1:1000 | Cell Signaling Technology/ Danvers, MA USA | 8025 |
| XIAP | Polyclonal | Rabbit | 1:2000 | Cell Signaling Technology/ Danvers, MA USA | 2042 |
