# Supplementary material for: Cancer Cell Growth Is Differentially Affected by Constitutive Activation of NRF2 by KEAP1 Deletion and Pharmacological Activation of NRF2 by the Synthetic Triterpenoid, RTA 405
Source: PLoS One. 2015 Aug 24;10(8):e0135257. doi: 10.1371/journal.pone.0135257 (PMC4547720; doi:10.1371/journal.pone.0135257)
Supplement: S3 Table — (DOCX) [file pone.0135257.s018.docx]

**Table S3. Effect of AIMs on Viability, Growth, and Apoptosis in Human Tumor Cell Lines**

| **Cell Line** | **Cancer Type** | **RTA 405 IC_50_^a^** | **RTA 402 IC_50_^b^** | **RTA 405 GI_50_^c^** | **RTA 405 Caspase-3,-7 Cleavage (%)^d^** |
| --- | --- | --- | --- | --- | --- |
| **Low Basal Nrf2 Activity** | | | | | |
| MG-63 | Osteosarcoma | 943.6 ± 112.3 | 361.2 (323.6 - 398.8) | 466.6 ± 79.1 | 9.1 ± 2.6 |
| BxPC-3 | Pancreatic adenocarcinoma | 1268.0 ± 77.8 | 247.5 (241.6 - 253.4) | 299.3 ± 48.0 | 47.6 ± 32.5 |
| PANC-1 | Pancreatic epithelioid carcinoma | 1073.0 ± 109.1 | 293.0 (292.2 - 293.8) | 830.7 ± 33.8 | 7.7 ± 3.6 |
| HCT 116 | Colorectal carcinoma | 509.8 ± 93.7 | 74.8 (65.6 - 84.0) | 406.4 ± 42.7 | 23.3 ± 2.4 |
| MDA-MB-231 | Breast adenocarcinoma | 806.6 ± 133.1 | 308.1 (261.5 - 354.7) | 497.6 ± 39.8 | 3.2 ± 0.9 |
| 786-0 | Renal cell adenocarcinoma | 448.7 ± 31.1 | ND | 392.1 ± 67.0 | 100.0 ± 0.0 |
| NCI-H23 | Lung (NSCLC) | 695.1 ± 33.8 | ND | 341.8 ± 34.6 | 10.1 ± 5.3 |
| SK-N-SH | Neuroblastoma | >1000 | ND | 393.8 ± 94.7 | 4.9 ± 1.8 |
| **Moderate Basal Nrf2 Activity** | | | | | |
| MCF-7 | Breast adenocarcinoma | 1272.0 ± 399.5 | 117.2 (99.0 - 135.4) | 382.6 ± 63.4 | 0.6 ± 0.6 |
| HT-29 | Colorectal adenocarcinoma | 591.2 ± 95.6 | 185.4 (171.4 - 199.3) | 416.9 ± 27.7 | 4.2 ± 1.1 |
| G-361 | Melanoma | 806.0 ± 141.2 | ND | 180.8 ± 38.6 | 30.2 ± 10.2 |
| HepG2 | Hepatocellular carcinoma | 940.9 ± 174.3 | 226.3 (206.3 - 246.3) | 316.3 ± 94.5 | 64.4 ± 13.4 |
| HCT-15 | Colorectal adenocarcinoma | 379.4 ± 46.8 | 106.2 (101.1 - 111.3) | 287.3 ± 12.1 | 43.9 ± 9.9 |
| **High Basal Nrf2 Activity** | | | | | |
| A2058 | Melanoma | 598.8 ± 80.2 | 162.2 (115.9 - 208.5) | 422.1 ± 25.4 | 15.1 ± 3.9 |
| SK-MEL-5 | Melanoma | >1000 | 146.1 (132.3 - 159.8) | 248.2 ± 4.8 | 47.3 ± 15.5 |
| HeLa | Cervical adenocarcinoma | 810.9 ± 88.9 | ND | 604.5 ± 176.8 | 11.0 ± 3.0 |
| A498 | Renal carcinoma | 713.6 ± 78.5 | ND | 432.8 ± 105.1 | 46.2 ± 8.4 |
| DU 145 | Prostate carcinoma | 654.6 ± 90.7 | ND | 376.4 ± 67.2 | 6.9 ± 2.0 |
| A549 | Lung carcinoma | 1142.0 ± 106.5 | 179.5 (150.5 - 208.6) | 647.0 ± 59.1 | 2.9 ± 1.2 |
| NCI-H460 | Lung carcinoma (NSCLC) | 335.1 ± 38.8 | 127.4 (126.2 - 128.5) | 289.4 ± 46.4 | 56.0 ± 6.5 |

a Values are mean and standard deviation of three independent experiments. Cells were treated for 48 hours

b Values are mean and range of two independent experiments. Cells were treated for 72 hours.

c Values are mean and standard deviation of three independent experiments. Cells were treated for 72 hours.

d Values are mean and standard deviation of three independent experiments. Values are shown as percent of caspase-3,-7 activation in 786-0 cells (set to 100%).
