# Supplementary material for: Cancer Cell Growth Is Differentially Affected by Constitutive Activation of NRF2 by KEAP1 Deletion and Pharmacological Activation of NRF2 by the Synthetic Triterpenoid, RTA 405
Source: PLoS One. 2015 Aug 24;10(8):e0135257. doi: 10.1371/journal.pone.0135257 (PMC4547720; doi:10.1371/journal.pone.0135257)
Supplement: S4 Table — (DOCX) [file pone.0135257.s019.docx]

**Table S4. Effect of RTA 405 on Markers of Apoptosis and Proliferation in Human Tumor Cell Lines**

|  |  | **Lowest Concentration of RTA 405 (nM) at Which Change in Marker Was Observed^a^** | | | | | | | | | | | |
| --- | --- | --- | --- | --- | --- | --- | --- | --- | --- | --- | --- | --- | --- |
|  |  | **Low Basal NRF2 Activity** | | | | **Moderate Basal NRF2 Activity** | | | | **High Basal NRF2 Activity** | | | |
|  |  | **n = 8** | | | | **n = 5** | | | | **n = 7** | | | |
| **Marker** | **Change** | **ND** | **250** | **500** | **1000** | **ND** | **250** | **500** | **1000** | **ND** | **250** | **500** | **1000** |
| **Caspase-3** | Cleavage | 3 (37.5) | 0 (0.0) | 1 (12.5) | 4 (50.0) | 3 (60.0) | 0 (0.0) | 0 (0.0) | 2 (40.0) | 2 (29.6) | 1 (14.3) | 1 (14.3) | 3 (42.9) |
| **Caspase-9** | Cleavage | 2 (25.0) | 0 (0.0) | 2 (25.0) | 4 (50.0) | 1 (20.0) | 0 (0.0) | 1 (20.0) | 3 (60.0) | 1 (14.3) | 1 (14.3) | 3 (42.9) | 2 (29.6) |
| **Cyclin D1** | Decrease | 0 (0.0) | 0 (0.0) | 3 (37.5) | 5 (62.5) | 0 (0.0) | 1 (20.0) | 2 (40.0) | 2 (40.0) | 1 (14.3) | 0 (0.0) | 0 (0.0) | 6 (85.7) |
| **CDKN1A (p21)** | Increase | 0 (0.0) | 4 (50.0) | 2 (25.0) | 2 (25.0) | 1 (20.0) | 2 (40.0) | 2 (40.0) | 0 (0.0) | 0 (0.0) | 5 (71.4) | 2 (29.6) | 0 (0.0) |
| **XIAP** | Decrease | 0 (0.0) | 1 (12.5) | 3 (37.5) | 4 (50.0) | 0 (0.0) | 0 (0.0) | 2 (40.0) | 3 (60.0) | 1 (14.3) | 0 (0.0) | 3 (42.9) | 3 (42.9) |
| **BIRC2** | Decrease | 1 (12.5) | 0 (0.0) | 1 (12.5) | 6 (75.0) | 2 (40.0) | 0 (0.0) | 1 (25.0) | 2 (40.0) | 2 (29.6) | 0 (0.0) | 2 (29.6) | 3 (42.9) |

a Number and percent of cell lines are shown

ND, Change not detected at concentrations tested
