# Supplementary material for: Cancer Cell Growth Is Differentially Affected by Constitutive Activation of NRF2 by KEAP1 Deletion and Pharmacological Activation of NRF2 by the Synthetic Triterpenoid, RTA 405
Source: PLoS One. 2015 Aug 24;10(8):e0135257. doi: 10.1371/journal.pone.0135257 (PMC4547720; doi:10.1371/journal.pone.0135257)
Supplement: S5 Table — (DOCX) [file pone.0135257.s020.docx]

**Table S5. *KRAS* Status in Human Tumor Cell Lines**

| **Cell Line** | **Cancer Type** | ***KRAS* status**^a^ |
| --- | --- | --- |
| **Low Basal Nrf2 Activity** | | |
| MG-63 | Osteosarcoma | WT |
| BxPC-3 | Pancreatic adenocarcinoma | WT |
| PANC-1 | Pancreatic epithelioid carcinoma | Mutant (G12D) |
| HCT 116 | Colorectal carcinoma | Mutant (G13D) |
| MDA-MB-231 | Breast adenocarcinoma | Mutant (G13D) |
| 786-0 | Renal cell adenocarcinoma | WT |
| NCI-H23 | Lung (NSCLC) | Mutant (G12C) |
| SK-N-SH | Neuroblastoma | WT |
| **Moderate Basal Nrf2 Activity** | | |
| MCF-7 | Breast adenocarcinoma | WT |
| HT-29 | Colorectal adenocarcinoma | WT |
| G-361 | Melanoma | WT |
| HepG2 | Hepatocellular carcinoma | WT |
| HCT-15 | Colorectal adenocarcinoma | Mutant (G13D) |
| **High Basal Nrf2 Activity** | | |
| A2058 | Melanoma | WT |
| SK-MEL-5 | Melanoma | WT |
| HeLa | Cervical adenocarcinoma | WT |
| A498 | Renal carcinoma | WT |
| DU 145 | Prostate carcinoma | Mutant (G12V) |
| A549 | Lung carcinoma | Mutant (G12S) |
| NCI-H460 | Lung carcinoma (NSCLC) | Mutant (Q61H) |

^a^ *KRAS* mutation information was obtained from Catalogue of Somatic Mutations in Cancer (COSMIC; <http://cancer.sanger.ac.uk/cancergenome/projects/cosmic/>), accessed 1/31/2014
